# Identifying the Growth Factors for Improving Neointestinal Regeneration in Rats through Transcriptome Analysis Using RNA-Seq Data

**DOI:** 10.1155/2018/4037865

**Published:** 2018-12-13

**Authors:** Shyh-Chuan Jwo, I-Fang Chung, Hsei-Wei Wang, Ting-Yu Chang

**Affiliations:** ^1^Division of General Surgery, Kaohsiung Municipal United Hospital, Kaohsiung, Taiwan; ^2^College of Pharmacy and Health Care, Tajen University, Pingtung County 90741, Taiwan; ^3^Division of General Surgery, Chang Gung Memorial Hospital, Keelung, and College of Medicine, Chang Gung University, Taoyuan, Taiwan; ^4^Division of General Surgery, Shin Kong Wu Ho-Su Memorial Hospital, Taipei, and School of Medicine, Fu Jen Catholic University, New Taipei City, Taiwan; ^5^Institute of Biomedical Informatics, National Yang-Ming University, Taipei, Taiwan; ^6^Center for Systems and Synthetic Biology, National Yang-Ming University, Taipei, Taiwan; ^7^Preventive Medicine Research Center, National Yang-Ming University, Taipei, Taiwan; ^8^Department of Medical Research, Changhua Christian Hospital, Changhua, Taiwan

## Abstract

Using our novel surgical model of simultaneous intestinal adaptation “A” and neointestinal regeneration “N” conditions in individual rats to determine feasibility for research and clinical application, we further utilized next generation RNA sequencing (RNA-Seq) here in normal control tissue and both conditions (“A” and “N”) across time to decipher transcriptome changes in neoregeneration and adaptation of intestinal tissue at weeks 1, 4, and 12. We also performed bioinformatics analyses to identify key growth factors for improving intestinal adaptation and neointestinal regeneration. Our analyses indicate several interesting phenomena. First, Gene Ontology and pathway analyses indicate that cell cycle and DNA replication processes are enhanced in week 1 “A”; however, in week 1 “N”, many immune-related processes are involved. Second, we found some growth factors upregulated or downregulated especially in week 1 “N” versus “A”. Third, based on each condition and time point versus normal control tissue, we found in week 1 “N” BMP2, BMP3, and NTF3 are significantly and specifically downregulated, indicating that the regenerative process may be inhibited in the absence of these growth factors. This study reveals complex growth factor regulation in small neointestinal regeneration and intestinal adaptation and provides potential applications in tissue engineering by introducing key growth factors identified here into the injury site.

## 1. Introduction

In this study, we used next generation RNA sequencing (RNA-Seq) and bioinformatics analyses to explore transcriptome changes in neoregeneration and adaptation tissue and to identify key growth factors affecting regeneration and adaptation of intestinal tissue. Our previous research reveals complex growth factor regulation in small intestine regeneration and adaptation [1] and provides potential applications in tissue engineering by introducing key growth factors identified here into the injury site. For this study we focused on our earlier research involving simultaneous intestinal adaptation and neointestinal regeneration in individual rats [1].

Treatment of patients with short bowel syndrome remains a challenge. Drugs, lengthening surgeries [2, 3], and total parental nutrition [3, 4] are problematical, and although developments in small bowel transplantation have made good progress, there are still donor shortages, immune rejection, and secondary malignancy induced by antirejection drugs [4, 5].

Regenerative processes occur in small bowel transplantation, intestinal adaptation [2, 6], and neointestinal regeneration for tissue engineering to regenerate a new small intestine [7]. Neointestinal regeneration, without a preexisting architecture, is improving as successful therapy for short bowel syndrome [2, 8] and addition of small segments of autologous grafts fabricated by tissue engineering is a compelling idea.

Using regenerative medicine, many organs and tissues are expected to be manufactured by tissue engineering [9–11]. Various experimental models for tissue-engineered neointestine have been developed [12–17], but no ideal model has yet been identified. Previous studies focused on either intestinal adaptation [2, 4, 18–21] or intestinal transplantation [22–25] alone. Our surgical model of simultaneous intestinal adaptation and neointestinal regeneration in individual rats was evaluated previously for its feasibility for future basic research and clinical application. There, a soft silastic tube was used as a tissue scaffold stent for neointestinal regeneration [1]. This model greatly reduces interexperimental variation and simplifies data interpretation. To facilitate neointestinal regeneration as well as have higher quality regenerative tissues, we aimed to understand the neointestinal regeneration mechanisms and then explore the biological functions of newly identified regeneration-related molecules in our rat model [1].

Intestine regeneration and adaptation are regulated by several classes of factors [26], such as nutritional factors, mesenchymal interactions, hormones, and growth factors [27]. Generally, growth factors, such as epidermal growth factor (EGF), are regarded as key factors for intestinal mucosal/epithelial homeostasis and regeneration [28–30]. While research indicates EGF's role in regeneration [27], as well as Wnt signaling, intestinal stem cells (ISCs) [28], and activation of a subset of Lgr5+ stem cells [29], nevertheless key growth factors that can initiate stem cell proliferation and differentiation during neointestinal growth remain subtle.

Therefore, in this study, based on our previous rat surgery model [18], we further utilized next generation sequencing to digitally measure the mRNA expression profile in both intestinal adaptation (abbreviated with “A”) and neointestinal regeneration (abbreviated with “N”) conditions across time to decipher the transcriptome changes in the neoregeneration tissues at weeks 1, 4, and 12 after surgery. As discussed below, our analyses indicate several interesting phenomena revealing complex growth factor regulation in intestinal adaptation and small intestine regeneration opening up potential pathways for effecting better applications for tissue engineering.

## 2. Materials and Methods

### 2.1. Data Source

#### 2.1.1. Animal

30 adult male Sprague-Dawley rats weighing 250-350 g were used for the present study. All animals were kept in a designated pathogen-free facility at the Animal Center of Keelung Chang-Gung Memorial Hospital in accordance with the rules and regulations of Institutional Animal Care and Use Committee (IACUC). This project was performed using the protocol approved by Keelung Chang-Gung Memorial Hospital IACUC # 2013112801. The animals were housed in individually ventilated cages (Allentown Micro-Vent System, Allentown, New Jersey, USA) in an air-conditioned room at 20-24°C (relative humidity 55±5%), with lights on from 7:00 am to 7:00 pm and given standard rat feed and water* ad libitum*. All animals were weighed and switched to oral glucose water 24 hours before surgery for tube implantation or sacrifice.

#### 2.1.2. RNA-Seq and Data Analysis

One microgram total RNA collected from rat small intestine was subjected to Illumina TruSeq RNA library construction. The sequencing libraries were barcoded and sequencing reaction was performed using Illumina HiSeq 2500 in National Yang-Ming University Genome Research Center. Raw sequence data was aligned to Rnor_6.0 genome with Ensembl release-85 gene annotation using TopHat v2.1.1 [30, 31]. The aligned reads were assembled according to Ensembl gene and transcript annotation using Cufflinks v2.2.1 [32], and the gene level read count matrix was loaded into DESeq2 [33] and Wald statistical testing was performed. Differential expressed genes with Benjamini-Hochberg false discovery rate (FDR) less than 0.05 and fold-change greater than 1.5 were considered significant. The flowchart of study design was demonstrated at Suppl. Fig.  1. The rat samples are listed in Table 1.

### 2.2. Surgical Model for Simultaneous Neointestinal Regeneration and Intestinal Adaptation in Rats

Rat surgery was performed by Dr. Jwo at the Chang Gung Memorial Hospital, Keelung [1]. In brief, following anesthetization using isoflurane inhalation (Abbott, USA), laparotomy was performed via upper median incision. The peritoneal cavity was exposed aseptically. After dividing the small bowel between the proximal jejunum 3 cm from the Treitz ligament and midgut 50% length of whole intestine, end-to-end jejunojejunostomy anastomosis was performed using 7-0 polypropylene sutures between both severed ends of proximal and distal jejunum to process adaptation segment and continue the intestinal integrity. To establish the neointestinal regeneration model, 0.5 cm donor of autologous intestine, accompanied by mesenteric vessels, was retrieved from the mid-portion of the severed intestinal tissue. Unnecessary redundant tissue was removed, with only 0.5 cm donor intestine being preserved and intubated by a 6 cm soft silastic Penrose tube for neointestinal regeneration. Donor intestinal segments were fixed upon the tube with 7-0 polypropylene sutures. An intraluminal tube served as a tissue scaffold and drained out through the abdominal wall via bilateral tube-enterostomies created in both flanks of the rats. Animals were sacrificed at 1, 4, and 12 weeks after surgery using isoflurane to anesthetization followed by CO_2_ inhalation for 5 min.

### 2.3. Histological Analysis

Newly regenerated constructs, along with the adapted segment of intestine, were harvested at weeks 1, 4, and 12 after surgery and normal intestine as control (day 0). The tissues were opened longitudinally, fixed in 10% buffered formalin, and embedded in paraffin. Specimens were sectioned onto slides 5 *μ*m in thickness and stained with hematoxylin and eosin (H&E staining) for microscopic examination of basic histological architecture. Histological analysis, including villus height, crypt depth, and newly regenerated length of neointestinal mucosa and muscle, was performed on each specimen using an Olympus IX-70 microscope accompanied by DP controller 3.2.1.276 calculation software. Villus height and crypt depth in neointestinal specimens were all measured in the mid-portion of each newly regenerated tissue. Common measures of 10 crypts/villus per animal were used to increase the validity.

### 2.4. Functional Annotation of Gene Sets

To study the biological function of stage specific upregulated genes, we utilized WebGestalt web server [34] to perform overrepresentation enrichment analysis (ORA) using Gene Ontology biological process category. Categories with at least five genes and with BH adjusted* P* value less than 0.05 were classified as significantly enriched. We also sent the differential expressed genes to Ingenuity Pathway Analysis (Qiagen, Germany) to perform network analysis and summarize the significant altered canonical pathways.

### 2.5. RNA Extraction and Reverse Transcription Quantitative PCR (RT-qPCR)

To validate the expression levels in sequencing data, total RNA samples from the intestines of rats were analyzed for mRNA expression by quantitative real-time RT-PCR (RT-qPCR). The 44 most significantly up- and downregulated genes were selected as candidate genes for validation. Primers for the target genes (Suppl.  Table 3) were designed with NCBI primer-BLAST and checked with Primer3. For mRNA, neointestine/adapted intestines of three rats at 0, 1, 4, and 12 weeks were harvested and frozen immediately with RNAlater reagent (QIAGEN, USA) in -80°C until further processing. Total RNA was extracted using* Direct-zol*™ RNA* MiniPrep* kit (ZYMO RESEARCH, USA) followed by reverse transcriptase using iScript cDNA synthesis kit (BioRed, USA). Target genes of primers were assessed by 2-step quantitative RT-PCR with SybrGreen Supermix (BioRed, USA) to label the amplified products. mRNA expression was quantified using the iCycler iQTM5 multicolor RT-qPCR detection system. Positive control was set with RNA alone and negative control without input of any templates. The relative expression levels of target genes were normalized against the expression level of GAPDH.

### 2.6. Statistics of Wet-Lab Validation

All data were expressed as the mean with standard deviation. The nonparametric Wilcoxon rank-sum test was used to test the difference among individual groups. All statistical analyses were performed using Statistical Package for Social Science version 17.0 (SPSS®, Chicago, Illinois, USA). A* P* value of less than 0.05 was considered statistically significant.

## 3. Results

### 3.1. Histological Analysis

**Figure 1:**
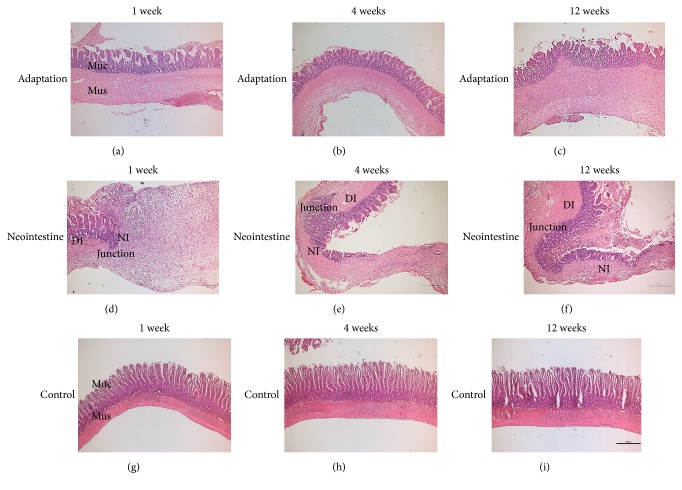
Histological analysis of regenerative constructs under various conditions. Hematoxylin and eosin staining of longitudinal sections of adapted intestine (a, b, c), regenerated neointestine (d, e, f), and the normal control (g, h, i) from weeks 1, 4, and 12 after surgery regenerative constructs. (a–c) Villi and crypts of tissues undergoing intestinal adaptation. (d–f) The regeneration process of neointestine was time-dependent. (g–i) No histological evidence of significant difference in normal control was identified at any time point. Muc, mucosa layer; Mus, muscle layer; DI, donor intestine; Junction; start point of regeneration; NI, neointestine. Magnification, 40X; bar, 500 *μ*m.

Figure 1 reflects the regeneration progress of intestinal adaptation over 12 weeks at 1, 4, and 12 weeks (Figures 1(a), 1(b), and 1(c)) and the progress of neointestine regeneration over 12 weeks at 1, 4, and 12 weeks (Figures 1(d), 1(e), and 1(f)). Comparisons of the two processes based on duration of regeneration for each process were ascertained microscopically (Figure 1). Adapted intestines more closely resembled normal control than those of regenerated neointestines, in particular mucosa thickness including villi and crypts, but this was not microscopically different among adapted intestines at each time point. In addition, muscle thickness of adapted intestines was more prominently time-dependent compared to that of normal control or regenerated neointestines (Figures 1(a), 1(b), and 1(c)). Neointestine regeneration was time-dependent (Figures 1(d), 1(e), and 1(f)), and in fact no histological evidence of regenerated neointestine was noted in the first week after surgery except severe inflammatory cell infiltration (Figure 1(d)). However, neointestinal specimens in the fourth week after surgery showed newly growing mucosa (and adhesive matrix with cryptogenesis), with progressive crypts accompanied by early evidence of muscular regeneration close to both ends of the donor intestine (Figure 1(e)). In the twelfth week after surgery of neointestinal specimens, the length of mucosa covering and dispersed muscular bundles increased dramatically compared to that of week 4 after surgery, inflammation subsided, and submucosa matrix remodeling beneath the neomucosa appeared with muscular bundles and fibroblasts (Figure 1(f)). Compared to intestinal adaptation, the tissue of regenerated neointestine showed thin, withered mucosa, obscure submucosal layer, disoriented muscle, and irregular serosal layers (Figures 1(a), 1(d), 1(e) and 1(f)).

### 3.2. *In Silico* Data Analysis

**Figure 2:**
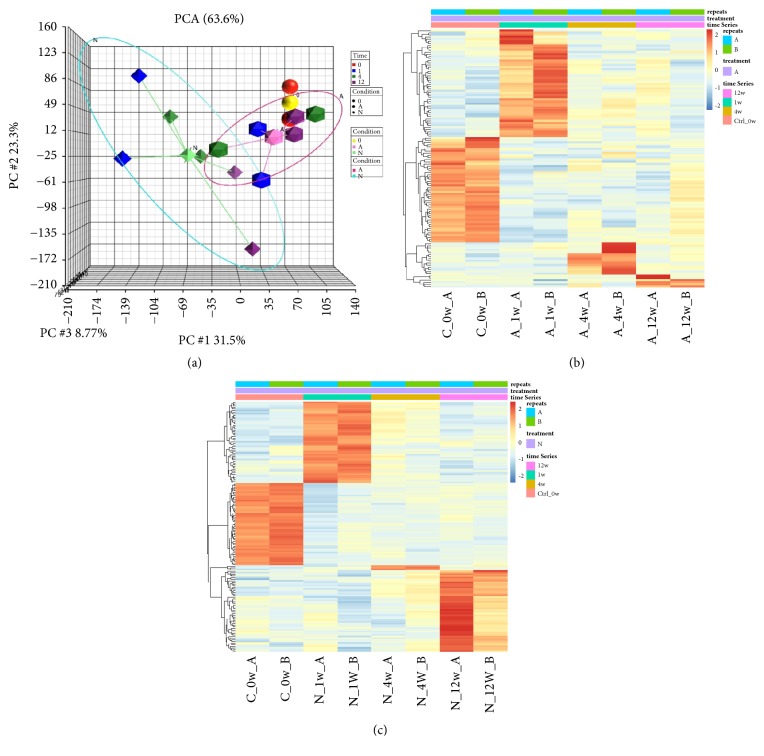
Global expression pattern of each condition and time point. (a) Principle component analysis (PCA) plot of each sample on whole transcriptome. Red: control; blue: week 1, green: week 4, purple: week 12. Octahedron: “N”, Icosahedron: “A”. Light green octahedron: centroid of “N”; pink icosahedron: centroid of “A”; yellow sphere: centroid of control. Blue oval: region of “N”; magenta oval: region of “A”. (b) Heat map of stage specific upregulated genes of “A”. (c) Heat map of stage specific upregulated gene of “N”.

To understand the underlying molecular mechanisms between “A” and “N” conditions over time, we performed RNA next generation sequencing at weeks 0, 1, 4, and 12 after surgery of both “A” and “N” conditions using Illumina HiSeq 2500 platform. First, we performed principle component analysis (PCA) across the whole transcriptome on each sample (Figure 2(a)). The x-axis represents PC1, with 31.5% variation; y-axis represents PC2, 23.3% variation; z-axis represents PC3, 8.77% variation. The icosahedral shape indicates samples were in “A”; the octahedral shape indicates “N”. The centroid of “A” was labeled with a pink icosahedral; the centroid of “N” was labeled with a light green octahedral; the centroid of control condition was labeled with a yellow sphere. The distribution of “A” was grouped by a magenta oval while “N” was grouped by a cyan oval. We can observe the control (0) condition locates close to “A”, especially at the 12-week. Both “A” and “N” of each group are considered as separate domains and all time points of “A” are more similar than “N”. Next, we tried to discover stage specific gene expression patterns in both conditions. Comparison of gene expression patterns between one time point versus all the others indicated both control (week 0) and week 1 had a greater number of up- or downregulated genes than weeks 4 and 12 did (Figures 2(b) and 2(c), detailed gene list in Suppl.  Dataset 1 and 2). We can also observe that genes specifically overexpressed in week 1 differentially clustered against control, week 4, and week 12, indicating that genes overexpressed in the first week may play distinct roles in tissue regeneration and remodeling.

### 3.3. Gene Expression between “A” and “N” Conditions at the Same Time Point

Next, we analyzed genes specifically upregulated in week 1 “A” by overrepresentation gene set analysis using the Gene Ontology database biological process section (Suppl. Tables 1 and 2). Only gene categories regarding mitotic cell cycle, DNA replication, and organic acid biosynthetic processes were significantly enriched in the gene set. On the contrary, genes specifically upregulated in week 1 “N” demonstrated several categories of immune response activation, chemotaxis, response to cytokine stimulus, and cellular component movement (Suppl.  Table 2). Moreover, we further analyzed the specific upregulated genes by Ingenuity Pathway Analysis and found that most of the genes specifically upregulated in week 1 “A” are related to biosynthesis and biogenesis of cholesterol, amino acids, folate, and oleate. Cell cycle and DNA replication pathways are also upregulated and transcription factor pathways including LXR/RXR RAN signaling are also upregulated. In week 1 “N”, pathways related to acute phase response signaling, IL-6, IL-10, IL-17A, and NF*κ*B signaling, Wnt/*β*-catenin, sonic hedgehog are all upregulated, indicating a state of inflammatory condition (Suppl. Fig.  2.)

Gene interaction subcellular network analysis on week 1 “N” indicates a network of cytokine/chemokine composed of IL1, IL10, IL1A, CXCL2, CXCL3, CXCL6, and TNFSF13 on the extracellular space, and a network of cytokine/chemokine/growth factor receptors composed of TNFRSF1A, CSCR2, TNFRSF9, TNFRSF21, TIE1, KDR, and BMPR2 on the plasma membrane (Suppl. Fig.  2). In the nucleus, the gene interaction network containing TWIST2, SNAI1, ABL1, and GSK3B, SMAD1 was also observed (Suppl. Fig.  2). Collectively, in week 1 “A”, tissue may undergo cell proliferation and biosynthesis of nutrients; on the other hand, in week 1 “N”, tissue may undergo an inflammation process and tissue remodeling including cell migration and angiogenesis. This molecular pattern in week 1 “N” may reflect a suppressed tissue regeneration and inflammatory phenotype as we observed in Figure 1(d).

**Figure 3:**
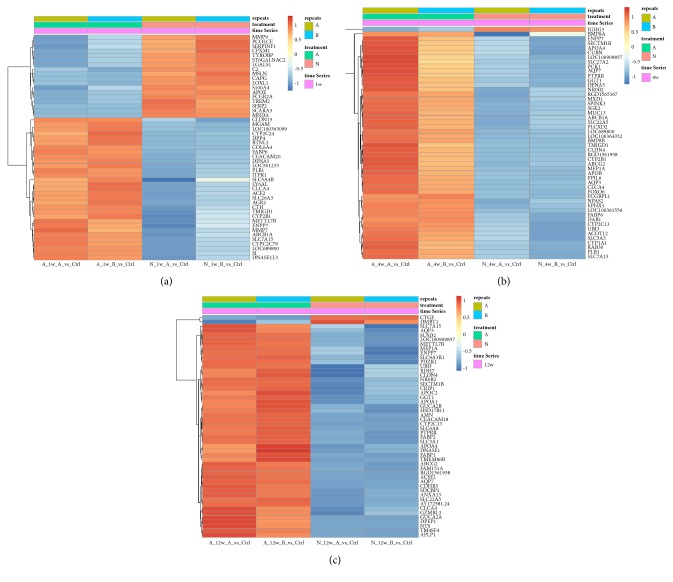
Gene expression pattern of each time point. (a) Heat map of the top 50 most significant genes of week 1 after surgical treatment for both “N” and “A” conditions. (b) Week 4. (c) Week 12.

Since stage specific overexpressed genes only give us limited information about the difference between two distinct surgical conditions (adaptive and neointestine), we then proposed another strategy to compare differential gene expression between these two conditions across three time points (weeks 1, 4, and 12). When statistical analysis was performed comparing “A” and “N” conditions at week 1, we found 1686 genes significantly upregulated and 1655 genes significantly downregulated in “N” (Figure 3(a), *P*_*adj*_ < 0.05, top 50 genes, gene list in Suppl.  Dataset 3). At week 4, we found 130 genes upregulated and 808 genes downregulated in “N” (Figure 3(b), *P*_*adj*_ < 0.05, top 50 genes, gene list in Suppl.  Dataset 3). At week 12, we found 1152 genes upregulated and 1993 genes downregulated in “N” (Figure 3(c), *P*_*adj*_ < 0.05, top 50 genes, gene list in Suppl.  Dataset 3). The gene sets at these three time points underwent Ingenuity Pathway Analysis, revealing in week 1 “N” pathways regarding hepatic fibrosis, atherosclerosis signaling, and granulocyte adhesion and diapedesis, together with the three most enriched pathways, again indicating a highly activated immune response and state of inflammation (Suppl. Fig.  3). On the contrary, in week 1 “A”, TCA cycle, IL-1 mediated inhibition of RXR function, PXR/RXR activation are enriched, suggesting a positive energy production for tissue remodeling/regeneration (Suppl.  Fig 3).

Interestingly, there are significantly fewer genes upregulated than downregulated in weeks 4 and week 12, indicating a relatively low transcription activity in weeks 4 and 12 “N”, especially in the top 50 most significant differential expressed genes (Figures 3(b) and 3(c)). This phenomenon is similar to the phenotype we observed in Figures 1(e) and 1(f). The tissue remodeling and regeneration activities arrested beginning in week 4 after operation; however, in week 4 “A” after operation, the regeneration process is still in progress. This may indicate in “N” that the microenvironment is not favorable for tissue regeneration and prone to an inflammatory state. At week 4, similar pathways again enriched in “A” including IL-1 mediated inhibition of RXR function, fatty acid beta-oxidation, PXR/RAR activation still become the top three on the list. However, there were too few genes specifically upregulated in week 4 “N” to perform pathway enrichment analysis.

**Figure 4:**
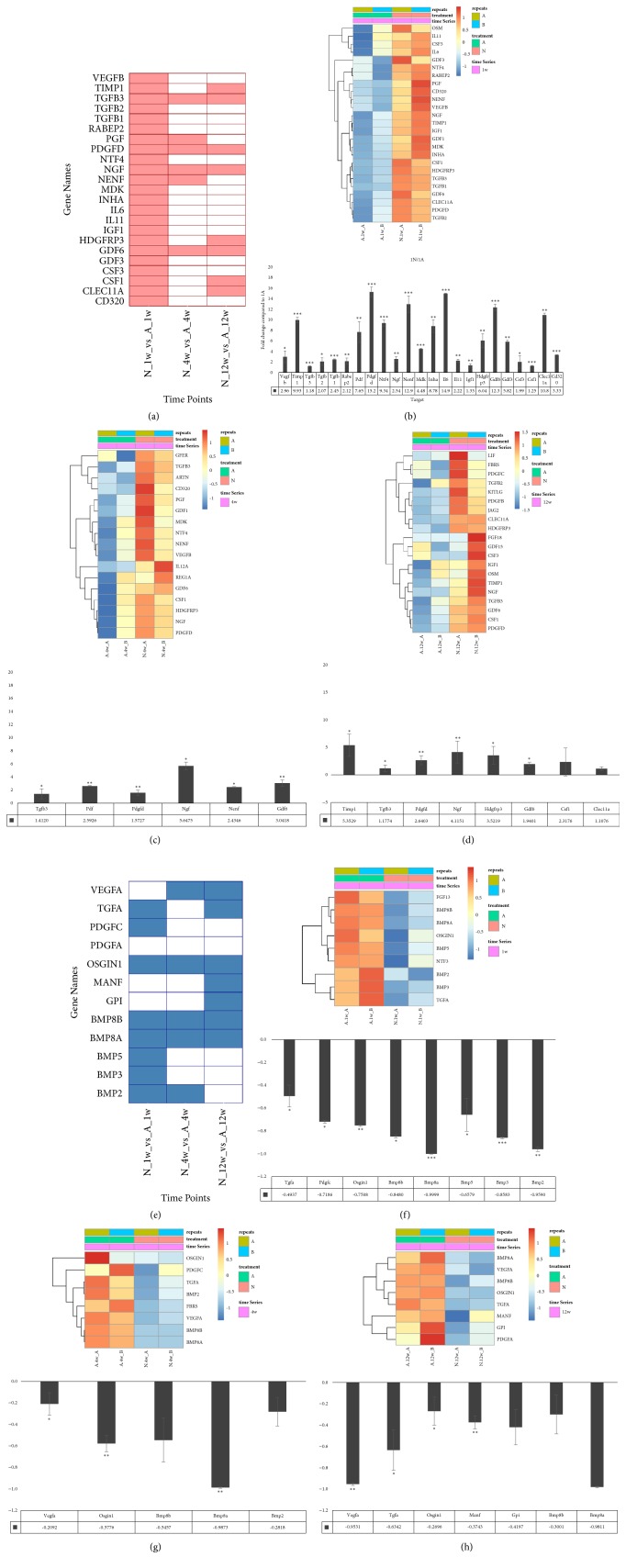
Growth factor expression patterns of each time point. (a) Tile chart demonstrated the significant upregulated growth factors in “N”. X-axis: “N” vs. “A” at week 1, week 4, and week 12. (b) Upper panel: heat map of differential expressed growth factors of both “N” and “A” at week 1. Lower panel: quantitative PCR validation of some growth factors. *∗∗∗*:* P* value < 0.001, *∗∗*:* P* value < 0.01, *∗*:* P* value < 0.05. (c) Week 4. (d) Week 12. (e) Tile chart for significant downregulated growth factors in “N”. (f) Upper panel: heat map of differential expressed growth factors of both “N” and “A” at week 1. Lower panel: quantitative PCR validation of some growth factors. (g) Week 4. (h) Week 12.

We therefore hypothesized that at week 1 “A”, critical growth factor combinations are required for proper tissue regeneration. We compared growth factor expressions among three different conditions at three time points, demonstrated as a tile chart in Figure 4(a). Red tiles indicate significant upregulated genes in “N” (*P*_*adj*_ < 0.05). For more comprehensive understanding of the expressions at each time point, we use heatmap to demonstrate differential expressed genes with unadjusted* P* value < 0.05 at the same time point. The upper panel of Figure 4(b) shows the expression profile of week 1, and the lower panel is qPCR confirmation of selected growth factors including RABEP2 (*P* < 0.001), TGFB1, GDF1, CD320 (*P* < 0.01) and VEGFB, MDK1 (*P* < 0.05). The differential expressed growth factor at week 4 and week 12 time points were also demonstrated (Figures 4(b) and 4(c), upper panel and Suppl.  Dataset 4) and confirmed by qPCR (Figures 4(b) and 4(c), lower panel). GFER and IL12A are significantly upregulated in week 4 “N” (*P* < 0.01) and REG1A (*P* < 0.05), whereas KITLG, JAG2, PDGFC, PDFGB, and FBRS are significantly upregulated in week 12 “N” (*P* < 0.05).

We then focused on growth factors upregulated in “A” across these same three time points. Data from RNA-Seq reveals another set of growth factors significantly upregulated in “A” at different time points. In Figure 4(e), blue tiles indicate significant upregulated growth factors in “A” (*P*_*adj*_ < 0.05.) The heatmap in the upper panel of Figures 4(f), 4(g), and 4(h) indicates the expression profile of growth factors (unadjusted* P* value < 0.05, detail data in Suppl.  Dataset 4). Quantitative PCR confirmed in week 1, BMP5 (*P* < 0.01), FGF13 and BMP3 (*P* < 0.05) are upregulated in “A” and in week 4 PDGFC (*P* < 0.01) and VEGFA (*P* < 0.05) (lower panels of Figures 4(f), 4(g) and 4(h)).

### 3.4. Gene Expression at Weeks 1, 4, and 12 for “A” and “N” Conditions Respectively Compared to Control

**Figure 5:**
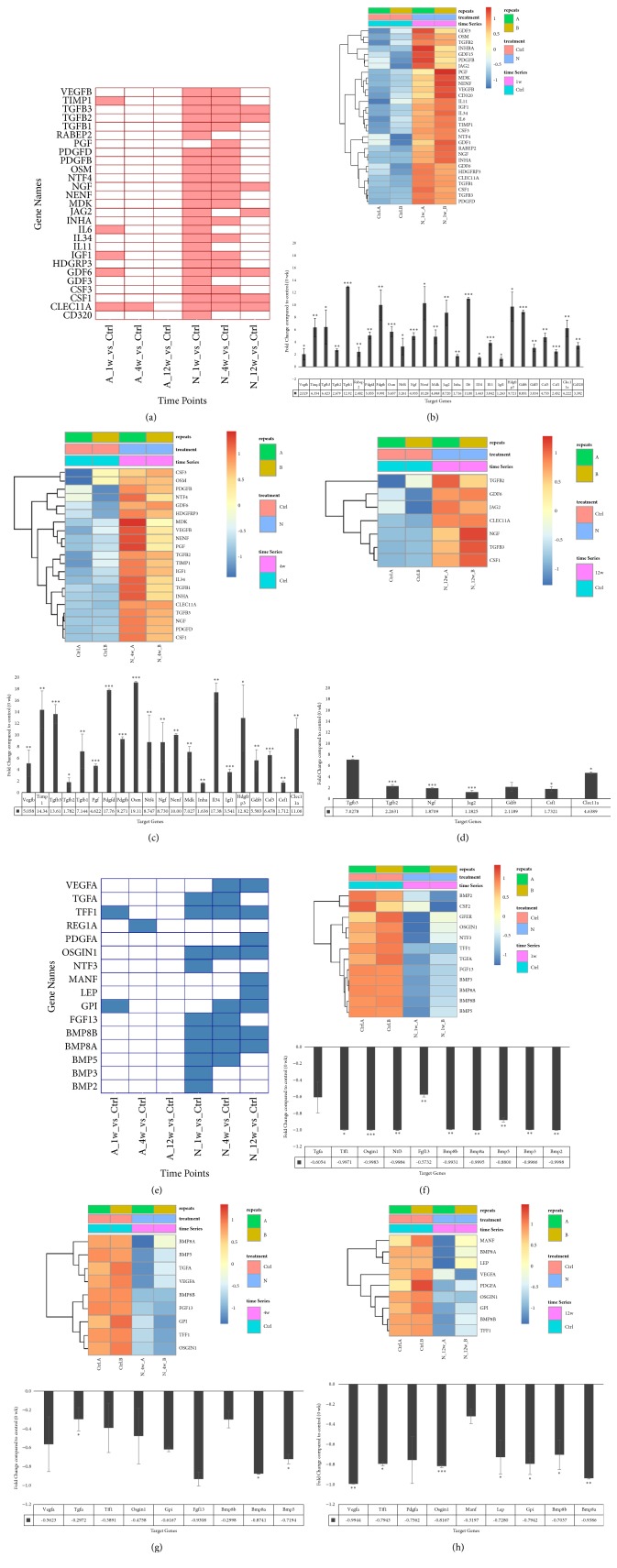
Stage specific growth factor expression patterns on A and N conditions. (a) Tile chart demonstrated the significant (*P*_*adj*_ < 0.05) upregulated growth factors in each time point and condition compared to control. (b) Tile chart demonstrated the significant (*P*_*adj*_ < 0.05) downregulated growth factors in each time point and condition compared to control. (c) Quantitative PCR confirmation of selected growth factors. (d) Heatmap of week 1 “A” and “N” compared to control. (e) Heatmap of week 1 “A” and “N” compared to control. (f) Quantitative PCR confirmation of selected growth factors.

Surprisingly, the number of growth factors upregulated in week 1 “N” is far more than in week 1 “A”. According to the microstructure demonstrated by FFPE H&E staining in Figure 1(a), we observed a proper stratified structure formed in the intestine adaptation specimen in week 1. However, in week 1 “N”, the majority part of the tissue was infiltrated with inflammatory cells (Figure 1(d)), indicating that many inflammation related cytokines, chemokines, and growth factors were highly expressed in this region, which may interfere with tissue regeneration. This also indicates “A” and “N” are very different in tissue composition. Condition “N” may not be a good baseline to pinpoint the key growth factors important for tissue regeneration in “A”. We then set the quiescent control tissue as a baseline for expression comparison and investigated the expression pattern of growth factors at different time points. We still observed many growth factors upregulated specifically in week 1 “N” (Figures 5(a) and 5(b)), consistent with our hypothesis. The number of the upregulation of growth factors was reduced in week 4 “N” and was even less in week 12 “N” (Figure 5(a)) indicating a decreasing inflammatory cell infiltration in the neoplastic site. This phenomenon is consistent with tissue microstructure (Figures 1(e) and 1(f)). In addition, several growth factors were also downregulated in weeks 1, 4, and 12 “N” (Figures 5(e), 5(f), 5(g), and 5(h)). On the contrary, only a few growth factors were downregulated in “A”: TFF1 and GPI in week 1, REG1A in week 4, and none in week 12. In week 1 “N”, BMP2, BMP3, BMP5, BMP8A, BMP8B, FGF13, NTF3, OSGIN1, TFF1, and TGFA were downregulated. This indicates that especially BMP signaling is repressed in early phases of neointestinal regeneration and may hamper the proper regeneration process.

## 4. Discussion

Transcriptome analysis on all time points after neointestine regeneration treatment and intestine adaptation treatment was carried out and gene expression profiles were compared either between the same time points or between the same conditions (“A” or “N”). After qPCR confirmation on different cohorts of rats, we identified highly expressed genes in week 1 “N”, including RABEP2, VEGFB, GDF1, IL-11, and CD320. These growth factors are correlated with inflammation after tissue injury [35], granulation tissue regeneration (VEGFB) [36], hematopoiesis (IL-11) [37], and neovasculature regeneration (VEGFB, RABEP2) [38], and most of them are originated from mesodermal tissue and may be regulated by GDF1 [39]. However, we identified highly expressed genes in week 1 “A”, including FGF13, NTF3, TFGA, BMP3, and BMP5. These factors have functions in promoting cell growth (FGF13) [40], supporting existing neuron survival neurotrophic factor (NTF3) [41], promoting small intestine crypt transit amplifying (TA) cell proliferation (TGFA) [42], and promoting existing TA cells differentiating into goblet cells (BMP3, BMP5) [43]. These genes belong to growth promotion of intestinal tissue. Moreover, in the first week of “N”, lower expression of BMP3 and BMP5 can derepress intestinal stem cells (ISC) in the crypt, therefore promoting intestinal regeneration. In week 4, genes which are highly expressed in “N”, including GFER, IL12A, and REG1A, play important roles in digestive organ regeneration, including GFER in liver regeneration [44], REG1A in pancreatic islet cells regeneration [45], and IL12A in antiangiogenesis [46]. On the contrary, in week 4 “A”, genes have functions related to mitosis, migration, and vasculogenesis (VEGFA) [47], maintaining and promoting connective tissue function and growth (PDGFC) [48]. In week 12 “A”, some growth factors upregulated including JAG2, PDGFC, OSM, PDGFB, and FBRS. These factors have roles in small intestine tissue regeneration, for instance, a ligand for intercellular signaling Notch pathway, JAG2 [49]; for growth and survival of connective tissue, PDGFC [48]; for stromal cell mitosis, proliferation smooth muscle cells, and endothelial cells in blood vessel (PDGFB) [50]; for proliferation of fibroblast (FBRS) [51]; for tumor suppression and liver development, angiogenesis, inflammation, and bone remodeling related factor oncostatin M (OSM) [52]. In week 12 “A”, there are no significant upregulated growth factors confirmed. Although in week 12 “N”, growth factors related to regeneration, for instance, JAG2, PDGFC, PDGFB, and FBRS, have higher expression rates than in “A”, some suppressive factors like OSM or other more potent factors repress the regeneration events in “N”. In addition, in week 12, the expression level of KITLG – a stem cell factor [53] in “N” is similar to “A”, indicating that the regeneration event is terminated for a while. This could explain why the regeneration activity in week 12 “N” is suppressed.

ISCs in the crypt base are maintained by their surrounding niche for precise regulation of self-renewal and differentiation under homeostasis [54, 55]. The ISC niche can be categorized fundamentally in the physical niche, and it refers to the extracellular matrix (ECM), which includes a multifunctional network of fibrous structural proteins (proteoglycans and glycoproteins) that act as scaffolding to maintain the three-dimensional architecture of the intestine [55]. Matrix proteins have been implicated in many cellular processes ranging from dynamic behavior such as migration and morphogenesis to cell-fate decisions such as proliferation, differentiation, and apoptosis [56]. The relationship between growth factors and the ECM is bidirectional. The ECM can regulate growth factor production and signaling, and growth factors can also alter the composition of the ECM [57]. Several growth factors play a prominent role in regulating the ECM, either by stimulating the production of ECM components or stimulating the production of molecules that break down the ECM [58]. Although PDGF and other growth factors are known to stimulate the production of structural ECM proteins such as collagen, TGF-*β*1 is one of the most important regulators of the ECM, regulating not only the production of multiple ECM components, but also collagen and fibronectin [59]. It also influences the ECM by inhibiting the production of proteases and increasing the synthesis of protease inhibitors [60]. The most investigated soluble factors that regulate the activation of the ECM-producing cells include cytokines [interleukin IL-6, IL-13, IL-17, IL-21, tumor necrosis factor TNF-*α*] [61], chemokines [monocyte chemotactic protein MCP-1, macrophage inflammatory protein MIP-1] [62], growth factors [transforming growth factor TGF-*β*1, connective tissue growth factor (CTGF), platelet derived growth factor (PDGF), insulin-like growth factor IGF-1 and IGF-2, epidermal growth factor (EGF)] [63], components of the renin-angiotensin system (RAS) [64], angiogenic factors [e.g., vascular endothelial growth factor (VEGF)] [65], peroxisome proliferator-activated receptors (PPARs) [66], mammalian target of rapamycin (mTOR) [67], and products of oxidative stress [35, 68]. Previous studies have identified several new SMAD gene targets among which are COL1A1, COL3A1, COL5A2, COL6A1, COL6A3, and TIMP-1 [69]. Most notably, the SMAD signaling pathway is crucial for simultaneous activation of several fibrillar collagen genes by TGF-*β* [70]. Besides playing a part in the regulation of the expression of ECM components, SMAD has recently been identified as capable of mediating the inhibitory activity of TGF-*β* on interstitial collagenase (matrix metalloproteinase-1) gene activation by proinflammatory cytokine [71, 72]. Interestingly, fundamental ECM factors, such as mechanical properties and biochemical signals that regulate ISCs colony and organoid formation, have recently been identified [73]. Communication between the ISCs and their niche is regulated by multiple signaling pathways such as the Wnt/*β*‐catenin cascade, Notch signaling, TGF‐*β*/bone morphogenic protein (BMP) pathways, and Hedgehog (Hh) pathways. There are two potential strategies to boost the neointestine regeneration process: one is to repress the overinfiltration of immunocytes into the regeneration site and another is adding the growth factors downregulated in week 1 “N” condition observed in Figure 5(b) into the scaffold to facilitate the proper cell growth and differentiation.

## Figures and Tables

**Table 1:** Rat samples collected for RNA-Seq.

	**Week 0**	**Week 1**	**Week 4**	**Week 12**
**Control**	2	0	0	0
**Intestinal adaption (A)**	0	2	2	2
**Neointestine regeneration (N)**	0	2	2	2
**Rat used**	6	6	6	6

## Data Availability

The data used to support the findings of this study are available from the corresponding author upon request.

## Supplementary Materials

Supplementary Materials Suppl. Table 1: Gene Ontology analysis on week 1 “A” condition upregulated genes. Suppl. Table 2: Gene Ontology analysis on week 1 “N” condition upregulated genes. Suppl. Table 3: Oligonucleotide sequences for qPCR validation of gene expression. Suppl. Figure 1: Flowchart of this study. Suppl. Figure 2: Stage specific genomic analysis for week 1 “N” or week 1 “A” conditions. Suppl. Figure 3: Stage specific genomic analysis for 1-, 4-, and 12-week “N” vs. “A” conditions. Suppl. Dataset 1: A_stage_specific.xlsx. Suppl. Dataset 2: N_stage_specific.xlsx. Suppl. Dataset 3: NvsA.xlsx. Suppl. Dataset 4: GF_time_matrix.xlsx.
