# Supplementary material for: Identifying the Growth Factors for Improving Neointestinal Regeneration in Rats through Transcriptome Analysis Using RNA-Seq Data
Source: Biomed Res Int. 2018 Dec 13;2018:4037865. doi: 10.1155/2018/4037865 (PMC6311312; doi:10.1155/2018/4037865)

Identifying the growth factors for improving neointestinal regeneration in rats through transcriptome analysis using RNA-Seq data

Short title: Identifying the growth factors for improving neointestinal regeneration

Shyh-Chuan Jwo[1,2,3,4,†,§], I-Fang Chung[5,6,7†], Hsei-Wei Wang[5], and Ting-Yu Chang[5,8,§]

[1]Division of General Surgery, Kaohsiung Municipal United Hospital, Kaohsiung, Taiwan. Postal address: No.976, Jhonghua 1st Rd., Gushan Dist., Kaohsiung City 804, Taiwan.

[2]College of Pharmacy and Health care, Tajen University, Pingtung County 90741, Taiwan. Postal address: No.20, Weixin Rd., Yanpu Township, Pingtung County 90741, Taiwan.

[3]Division of General Surgery, Chang Gung Memorial Hospital, Keelung, and College of Medicine, Chang Gung University, Taoyuan, Taiwan. Postal address: No. 222, Maijin Road, Anle District, Keelung City, 204, Taiwan.

[4]Division of General Surgery, Shin Kong Wu Ho-Su Memorial Hospital, Taipei, and School of Medicine, Fu Jen Catholic University, New Taipei City, Taiwan. Postal address: No. 95, Wenchang Road, Shilin District, Taipei City, 111, Taiwan

[5]Institute of Biomedical Informatics, National Yang-Ming University, Taipei, Taiwan. Postal address: No. 155, Sec 2, Li-Nong St., Peitou Dist. Taipei City, 11221, Taiwan

[6]Center for Systems and Synthetic Biology, National Yang-Ming University, Taipei, Taiwan. Postal address: No. 155, Sec 2, Li-Nong St., Peitou Dist. Taipei City, 11221, Taiwan

[67]Preventive Medicine Research Center, National Yang-Ming University, Taipei, Taiwan. Postal address: No. 155, Sec 2, Li-Nong St., Peitou Dist. Taipei City, 11221, Taiwan

[8]Department of Medical Research, Changhua Christian Hospital, Changhua, Taiwan. Postal address: No. 135, Nan-Hsiao St., Changhua City, Changhua County, 50006, Taiwan

†: These 2 authors contributed equally to this work

§: To whom correspondence to be addressed.

E-mail of all authors:

Jwo, Shyh-Chuan: jwosc1@gmail.com

Chung, I-Fang: ifchung@ym.edu.tw

Wang, Hsei-Wei: hwwang@ym.edu.tw

Chang, Ting-Yu: 182451@cch.org.tw

Corresponding authors:

Ting-Yu Chang, Ph.D.

Department of Medical Research, Changhua Christian Hospital, Changhua, Taiwan.

e-mail: 182451@cch.org.tw; phone: 886-4-723-8595 ext.2325; fax: 886-4-7232942

Postal address: No. 135, Nan-Hsiao St., Changhua City, Changhua County, 50006, Taiwan.

Shyh-Chuan Jwo, MD, Ph.D.

Division of General Surgery, Kaohsiung Municipal United Hospital, Kaohsiung, Taiwan.

Postal address: No.976, Jhonghua 1st Rd., Gushan Dist., Kaohsiung City 804, Taiwan.e-mail: jwosc1@gmail.com ; phone: 886-7-555-2565 ; fax: 886-7-555-3984

†: These 2 authors contributed equally to this work

§: To whom correspondence to be addressed.

Supplementary information

Suppl. Table 1: Gene Ontology analysis on 1-week A condition up-regulated genes

Suppl. Table 2: Gene Ontology analysis on 1-week N condition up-regulated genes

Suppl. Table 3: Oligonucleotide sequences for qPCR validation of gene expression

Suppl. Figure 1: Flowchart of this study

Suppl. Figure 2: Stage specific genomic analysis for 1-week N or 1-week A conditions

Suppl. Figure 3: Stage specific genomic analysis for 1-, 4-, and 12-week N vs. A conditions

Suppl. Table 1. Gene Ontology analysis on 1-week A condition up-regulated genes

GO:0007049	cell cycle
GO:0000278	mitotic cell cycle
GO:0022402	cell cycle process
GO:0044283	small molucule biosynthetic process
GO:0006261	DNA-dependent DNA replication
GO:0006259	DNA metabolic process
GO:1903047	mitotic cell cycle process
GO:0016053	organic acid biosynthetic process
GO:0046394	carboxylic acid biosynthetic process
GO:0044711	single-organism biosynthetic process
GO:0006260	DNA replication
GO:0009396	folic acid-containing compound biosynthetic process
GO:0007067	mitotic nuclear division

Suppl. Table 2. Gene Ontology analysis on 1-week N condition up-regulated genes

GO:0006952	defense response
GO:0002376	immune system process
GO:0016477	cell migration
GO:0060326	cell chemotaxis
GO:0006954	inflammatory response
GO:0008283	cell proliferation
GO:0007166	cell surface receptor signaling pathway
GO:0070887	cellular response to chemical stimulus
GO:0048870	cell motility
GO:0051674	localization of cell
GO:0006955	immune response
GO:0042127	regulation of cell proliferation
GO:0050900	leukocyte migration
GO:0048584	positive regulation of response to stimulus
GO:0030595	leukocyte chemotaxis
GO:0010562	positive regulation of phosphorus metabolic process
GO:0045937	positive regulation of phosphate metabolic process
GO:0032496	response to lipopolysaccharide
GO:0097529	myeloid leukocyte migration
GO:0009605	response to external stimulus
GO:0040011	locomotion
GO:0034097	response to cytokine
GO:0071345	cellular response to cytokine stimulus
GO:0002237	response to molucule of bacterial origin
GO:0002682	regulation of immune system process
GO:0001568	blood vessel development
GO:0006928	movement of cell or subcellular component
GO:0097530	granulocyte migration
GO:0001944	vasculature development
GO:0051239	regulation of multicellular organismal process
GO:1990266	neutrophil migration
GO:0072358	cardiovascular system development
GO:0009617	response to bacterium
GO:0006935	chemotaxis

GO:0042330	taxis
GO:0001816	cytokine production
GO:0071621	granulocyte chemotaxis
GO:0002684	positive regulation of immune system process
GO:0048514	blood vessel morphogenesis
GO:0071310	cellular response to organic substance

Suppl. Table 3. Oligonucleotide sequences for qPCR validation of gene expression

Gene	Forward	Reverse
GAPDH	GTGGCTGGCTCAGAAAAAGG	GGGGAGATTCAGTGTGGTGG
VEGFB	CAGCCATCAAAGCAGAATCA	ATGTAGGTGGGTGCCTGAAG
TIMP1	TGCAGAACATTGGCTTTCTG	ATGGCTGAACAGGGAAACAC
TGFB3	CTCTCTGTCCACTTGCACCA	GTTTCCTGAGTGCAGCCTTC
TGFB2	CCACACACTCTTTCCCTGGT	TTTCCATCACCCCCATAAAA
RABEP2	GGGAGCAACTGCAGGTAGAG	CACAGGCTAGGCAAGTCCTC
PDF	GAGGTGTGTGGGACTGAGGT	CAGGAGGAGGCACTCAACTC
PDGFD	GGGTATCGGGTTCTTTCCAT	TTCCTCGGAAATTGGACTTG
NTF4	CACTGGCTCTCAGAATGCAA	GTAGAGCAGTCGAGCCATCC
NGF	CCACCAACTGGAGACCAAGT	TTGTCGTGGTCATGGTGTCT
NENF	TGGAGGGCAGACAGAGAAGT	TGAGGAAGTCACTGGTGCAG
MDK	AGCCTGGAAGGAAGGTTTGT	TAGCAGCCCCAGCACTCTAT
INHA	GCTCTACCAGGGAGCATGAG	CACCTTCCTCCTAGCTGACG
IL6	CCAGGGCATTTTCCAGTTTA	ACTGGCATGGGGACTAACAG
IL11	GTAGCACATGGTGGTTCACG	GCAACACATGGTGGCTTATG
IGF1	GCACACACAGCCTGAAAAGA	GAGACTTGCCTTTGGCAGAC
HDGFRP3	CCCTGCAGTCACCACCTTAT	CAGACAGGTCCCTTGAGAGC
GDF6	TTGAACCTTTGCCTTCTGCT	AAAAAGCTCAGCCAGTGGAA
GDF3	GAGAATGGCCTTGAGAGTGC	GTCCTGGTACCTGGGCTGTA
CSF3	CCTTGGAGCAAGTGAGGAAG	GTGCCCCATGTTATGTTTCC
CSF1	GCAAGCAAGCAAAACATTCA	TTCCTCTCCCTTCCCAGAAT
CLEC11A	AGATGGGACAGTGGGTTCAG	CCAGACCTCTTTGCCTTCAG
CD320	GCAAGGGCTCTACCACTGAG	ATGTTCCAGGGTGCAGAAAC
VEGFA	TGCTTCCTAGTGGGCTCTGT	GATGCGAATCCTTTCCAAAA
TGFA	GCAAGTTCTGCCTGTTCCTC	GCACTGAACCAACCCACTTT
PDGFC	AACGTGTTCTCCTCCACACC	AGCTCTGCCCAAGACCTGTA
OSGIN1	CCCAGGAGCCTGTGTGTATT	TCCTGCTTCCTTGTTTGCTT
MANF	TGCAGACAAGCAGATTGACC	AGCAGGAACTGGGCAGACTA
GPI	GCAGAGGAGGGTTCTCTGTG	GGCGGTCACACCTGTAATCT
BMP8B	AGGACATAGGGAACCCATCC	TCTGCTCTTCCTGGGACACT
BMP8A	GGACAGTGGGGAGGTACAGA	ACAGAGGCTTGGGTGCTCTA
BMP5	GTACATGGCACGGAGGAAGT	ACAAACGCAGCCCAAATAAC
BMP2	AGGGTCTAGGGGATGTTGCT	CTCACATCTGCTTGCCAAAA
TGFB1	CTGGAGGCTGAGGCAATAAG	AGAAGGCAGAGGTGAGTGGA

PDGFB	GGAGGTCACTGCTGCTTTTC	GACTTTCCTTGGAGCTGTGC
OSM	GTTTCAAAAACGGGCAGTGT	CCACTATGAGCCCAGGTTGT
JAG2	ATTGGGGGATTTGTCTAGGG	CATCCCAGCAGTCAGAGTGA
IL34	GGAGGGGTGGCAAAATAAAT	TCTCCACTGCCACTTCCTCT
TFF1	CACCAAGGAGCCAATTCCTA	TTGAAAGGGCAACAAATTCC
REG1A	GCAATGTCAGTCATGGGTTG	GCCTGCCCAAATCTTTCATA
PDGFA	ACCCCTGCTTCAGTCTCTCA	AGAAGCCTCACCTCTGTCCA
NTF3	GCTCAACGAAAGCCAAAGTC	ACAGAACCACAGCCCGTATC
LEP	TGGCTCTTCTGCTTTTGGTT	CCCAACAGCACATAACATGC
FGF13	GCATGGGTGTTTAGGCAAGT	ATCCTAAGCGTAGCCAGCAA
BMP3	CCGAAATCCAATCTTCCTCA	AGAGGAGCCAGTGGTCAGAA

S1 Flowchart of this study

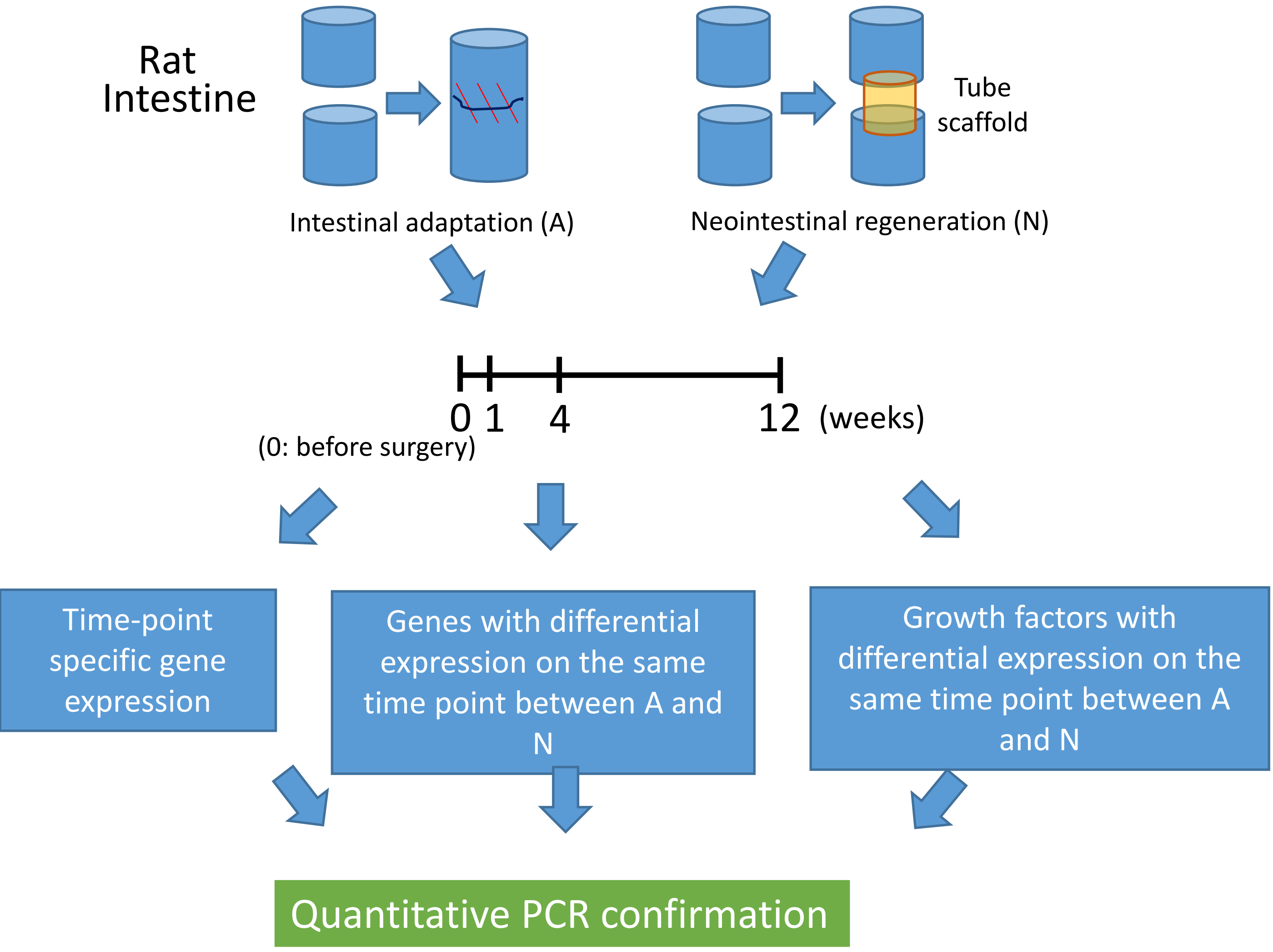

S2A Stage specific canonical pathway week 1 N condition

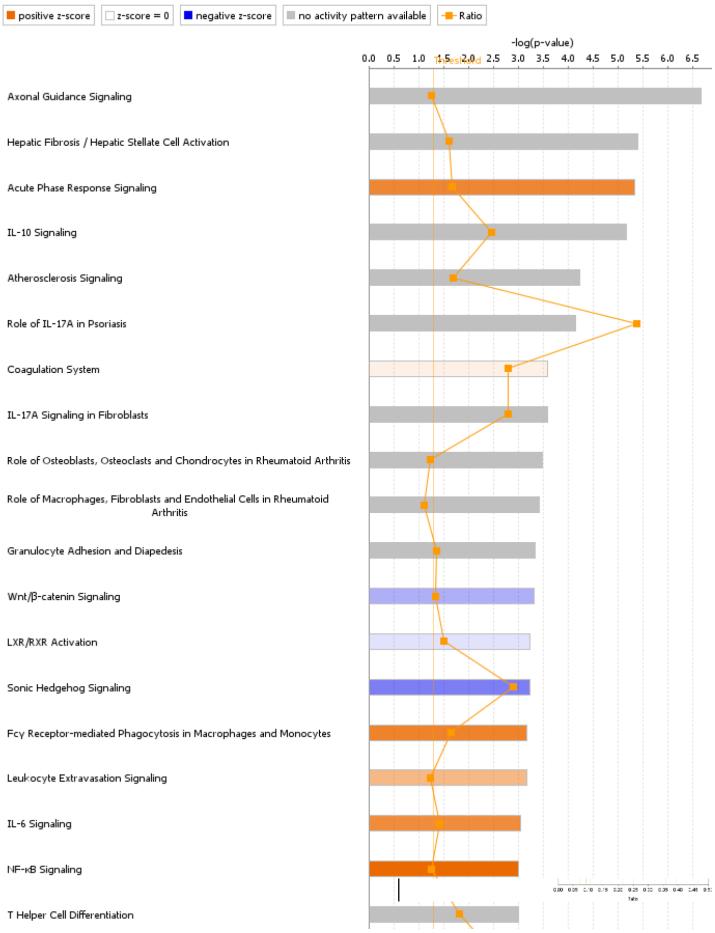

S2B Stage specific gene interaction network week 1 N condition

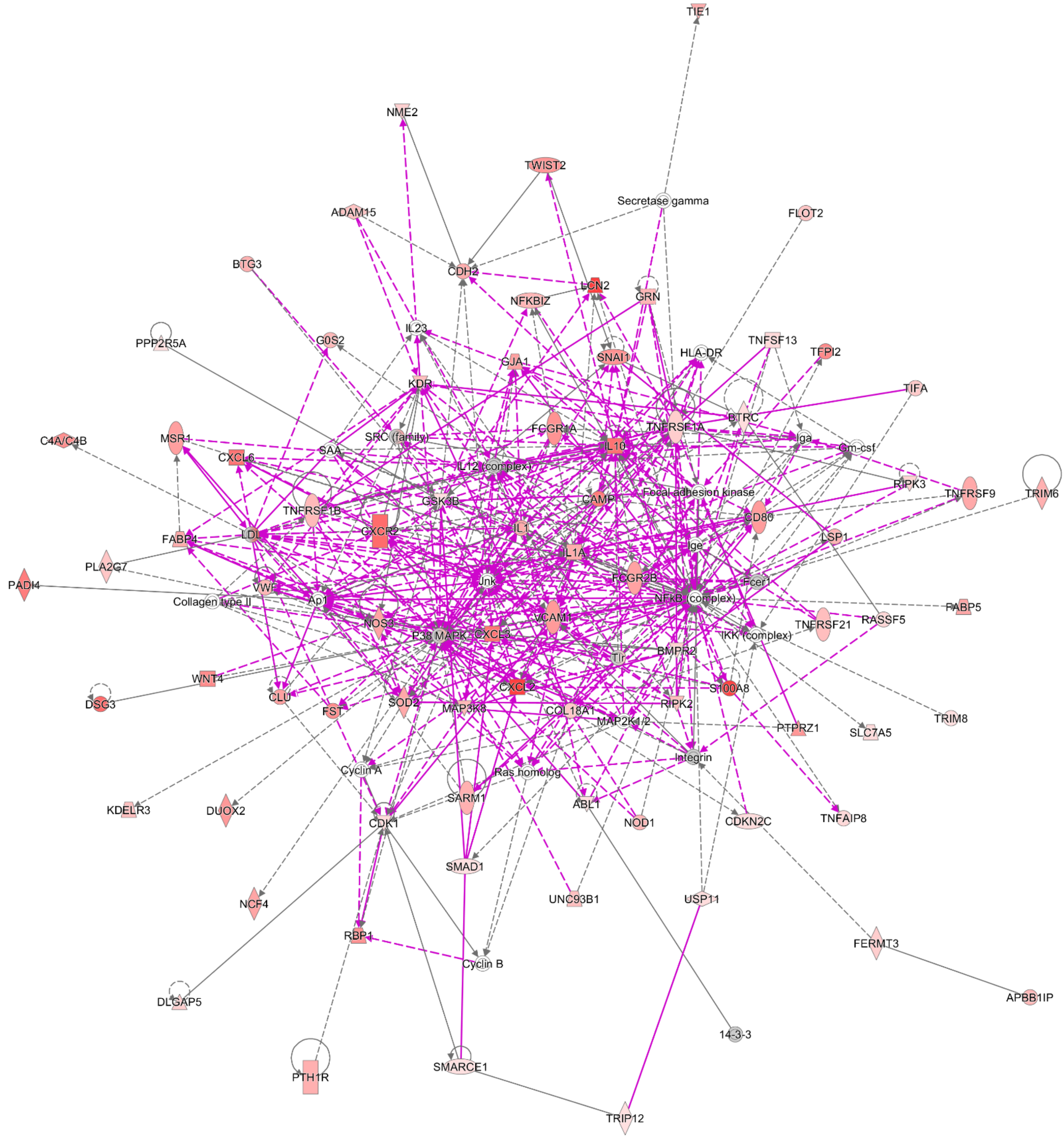

© 2000-2017 QIAGEN. All rights reserved.

S2C Stage specific gene interaction network with sub-cellular localization week 1 N condition

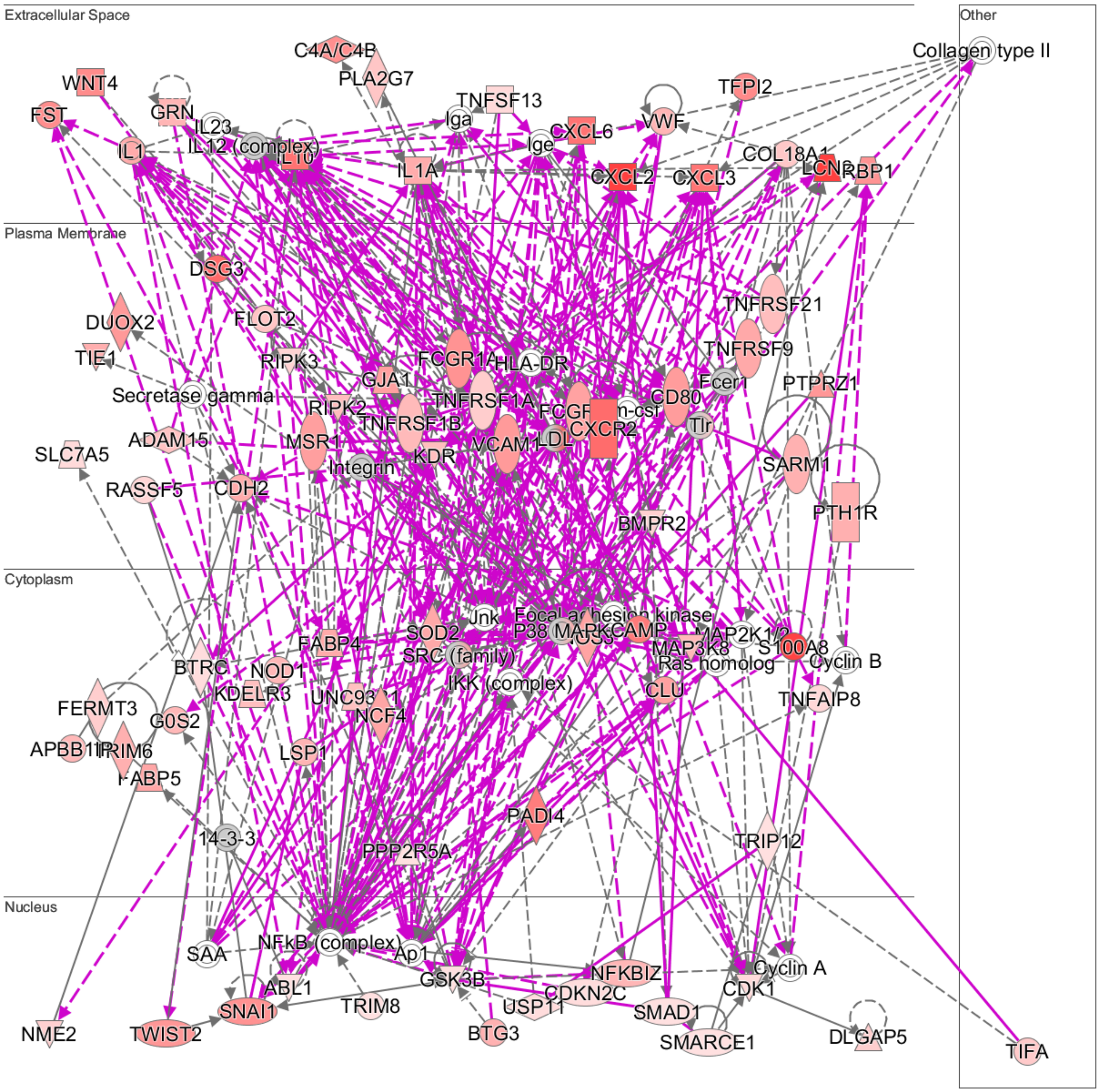

© 2000-2017 QIAGEN. All rights reserved.

S2D Stage specific Gene Ontology analysis

week 1 N condition

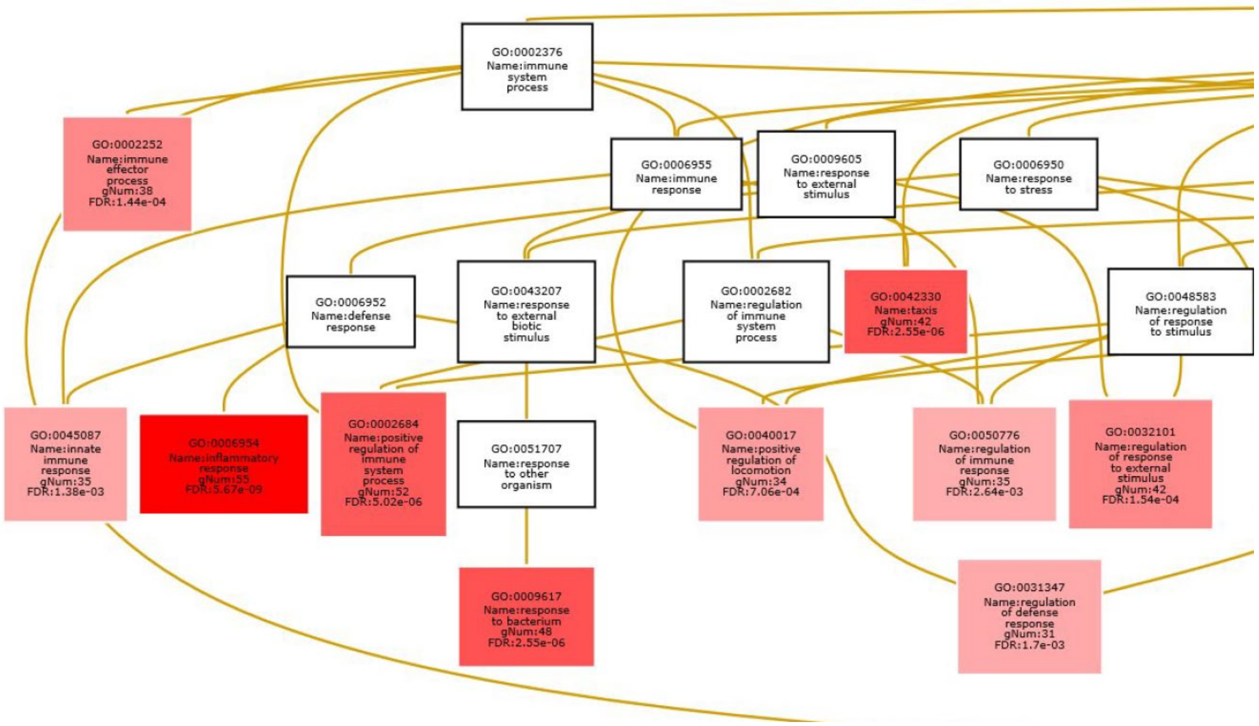

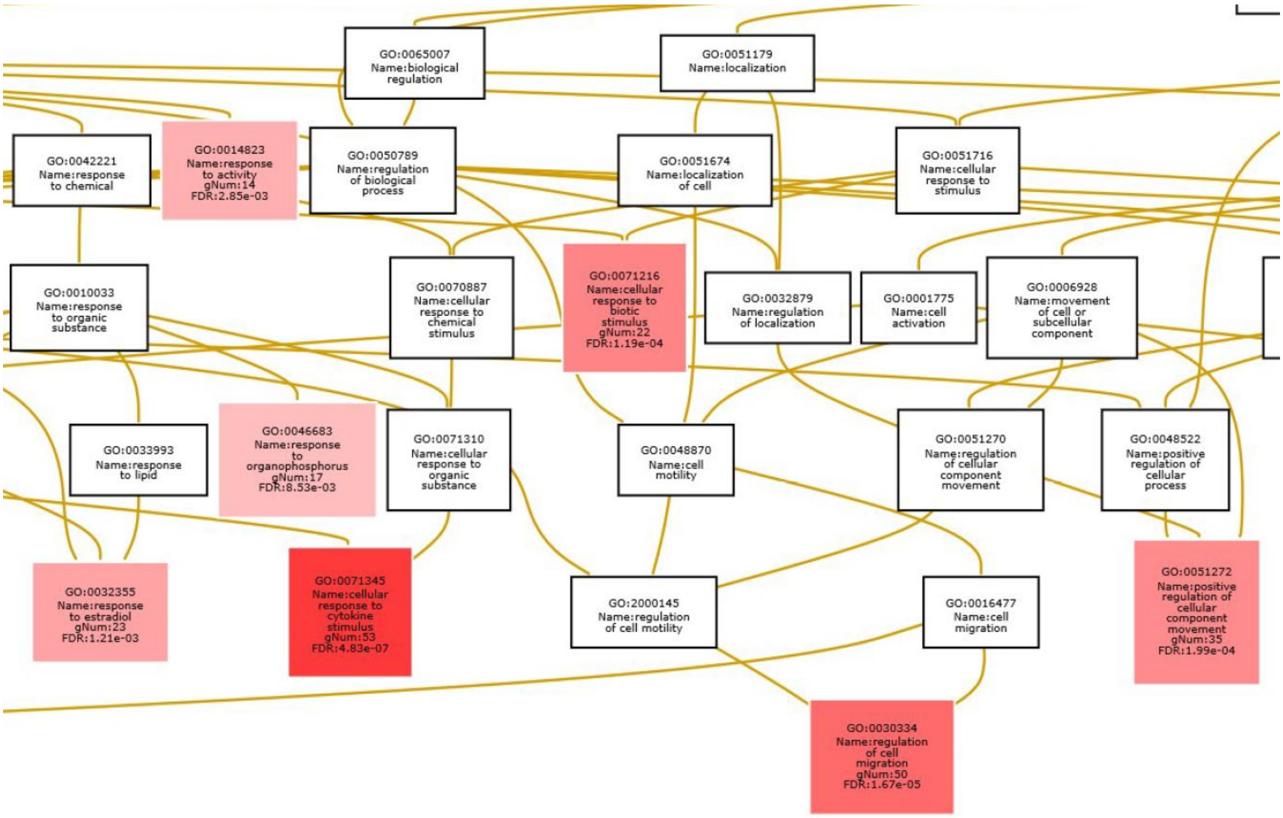

S2E Stage specific canonical pathway week 1 A condition

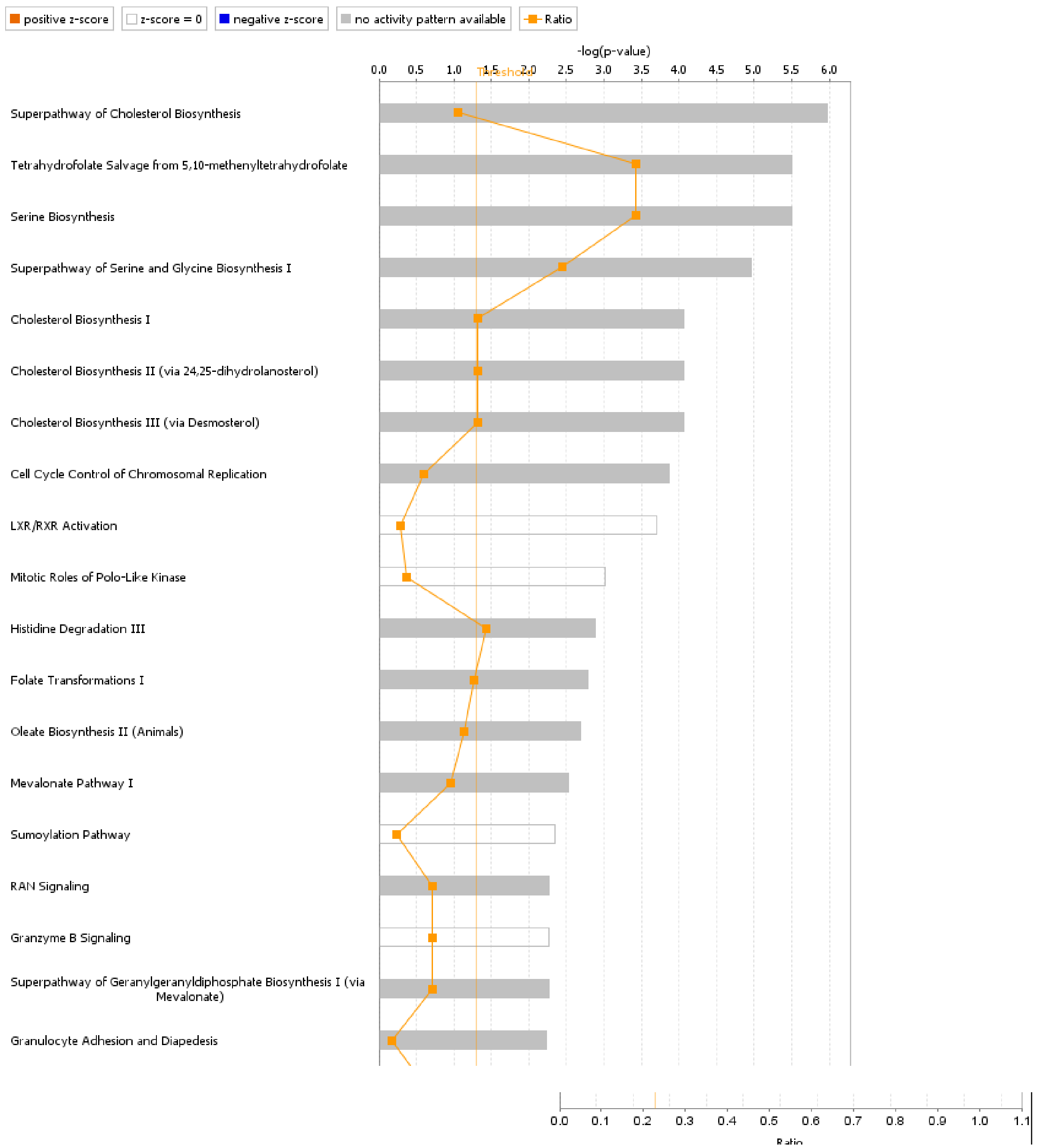

S2F Stage specific Gene Ontology analysis

week 1 A condition

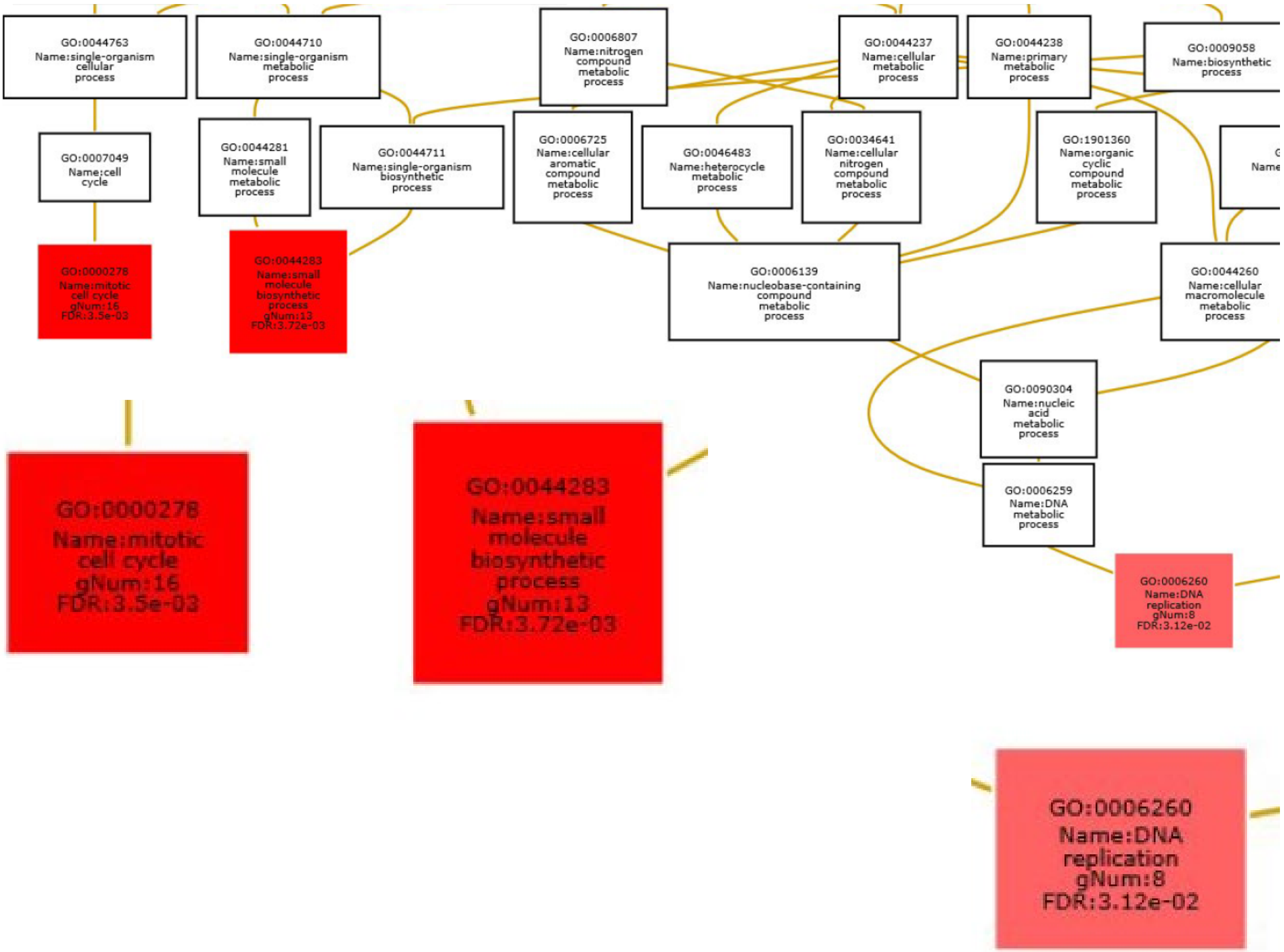

S2G Stage specific gene interaction network week 1 A condition

Networks 2,4,5 Merged 1

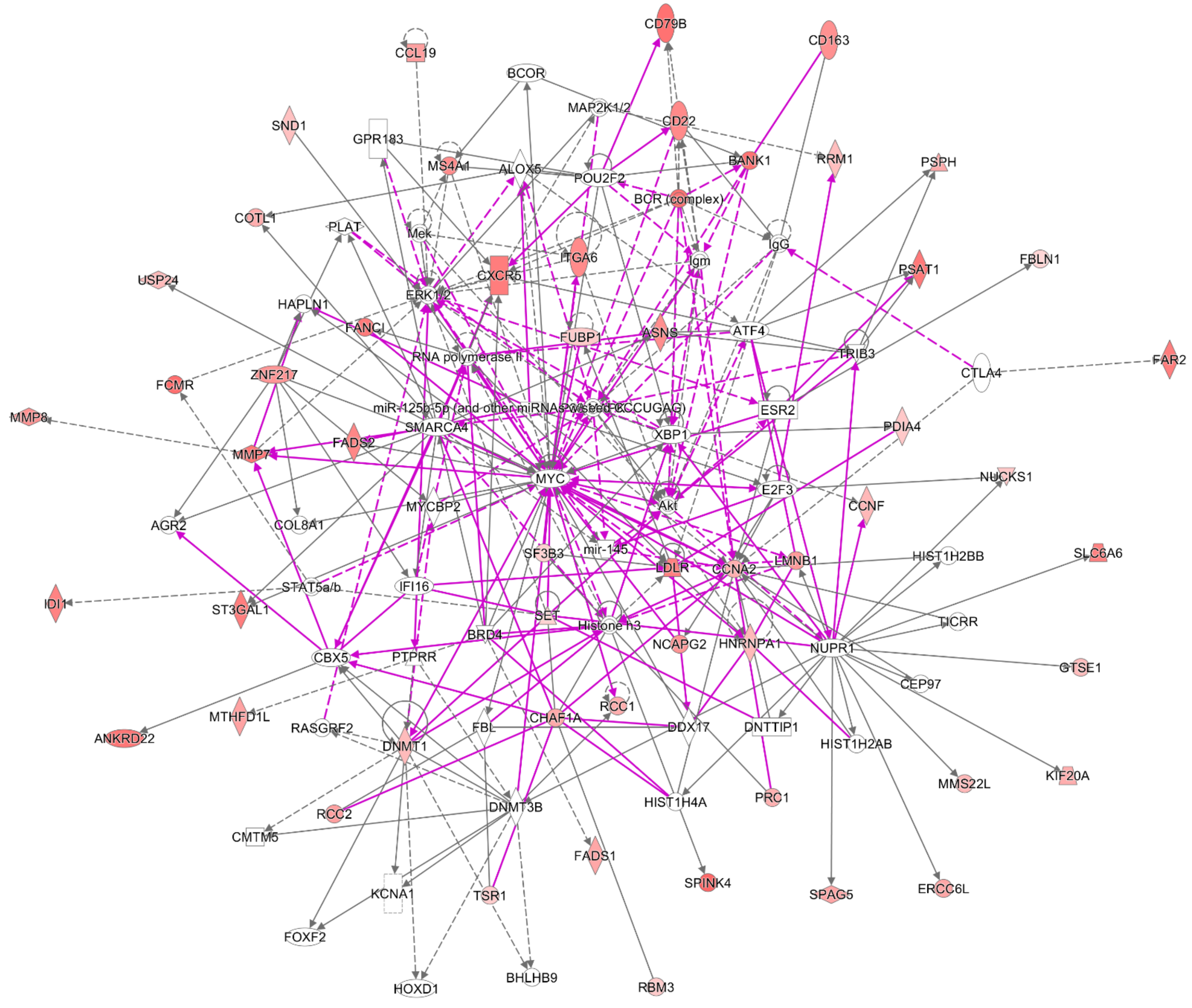

© 2000-2017 QIAGEN. All rights reserved.

S2H Stage specific gene interaction network with sub-cellular localization week 1 A condition

Networks 2,4,5 Merged 1

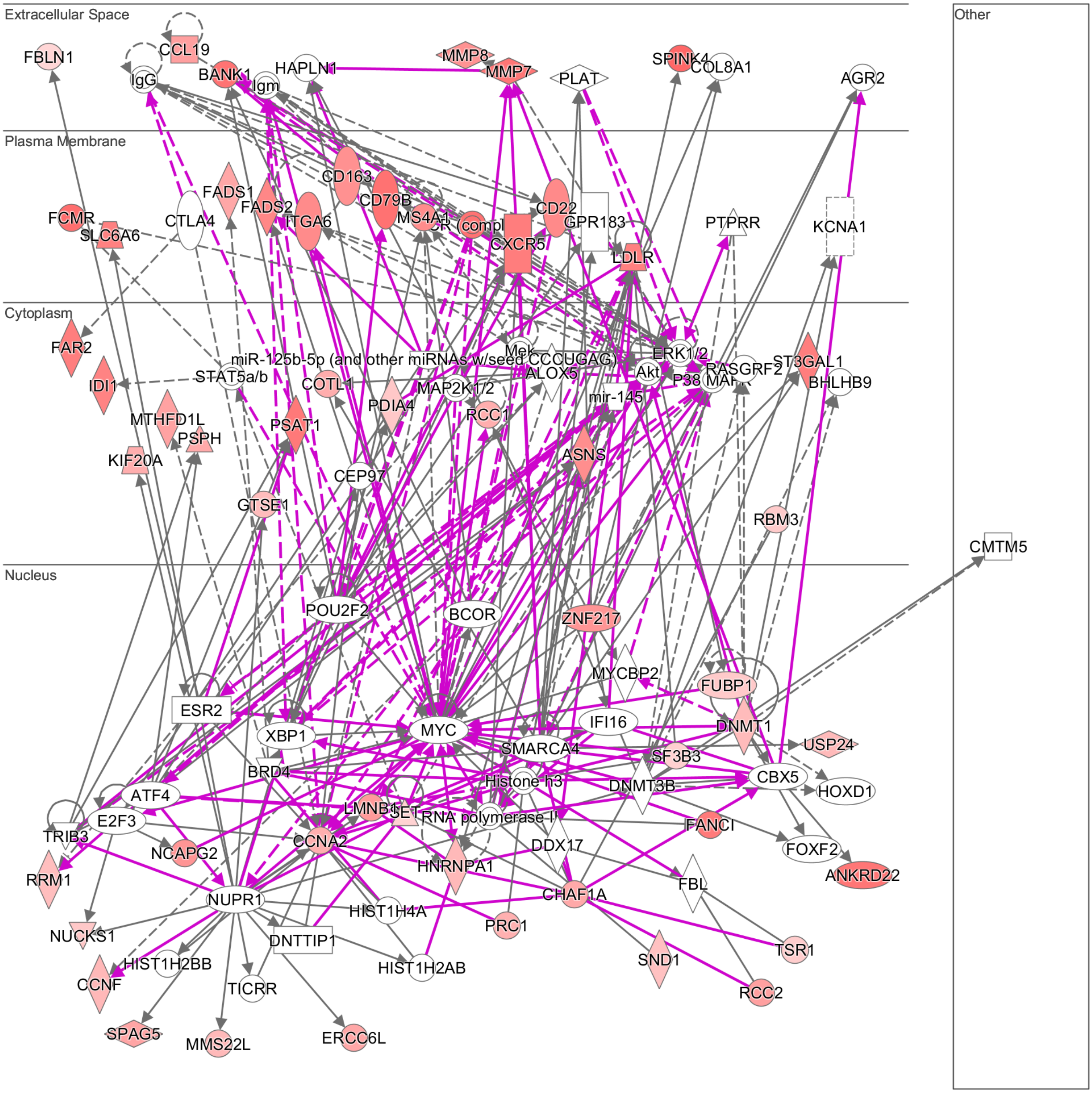

© 2000-2017 QIAGEN. All rights reserved.

S3A Stage specific canonical pathway
week 1 N vs week 1 A condition, N upregulated

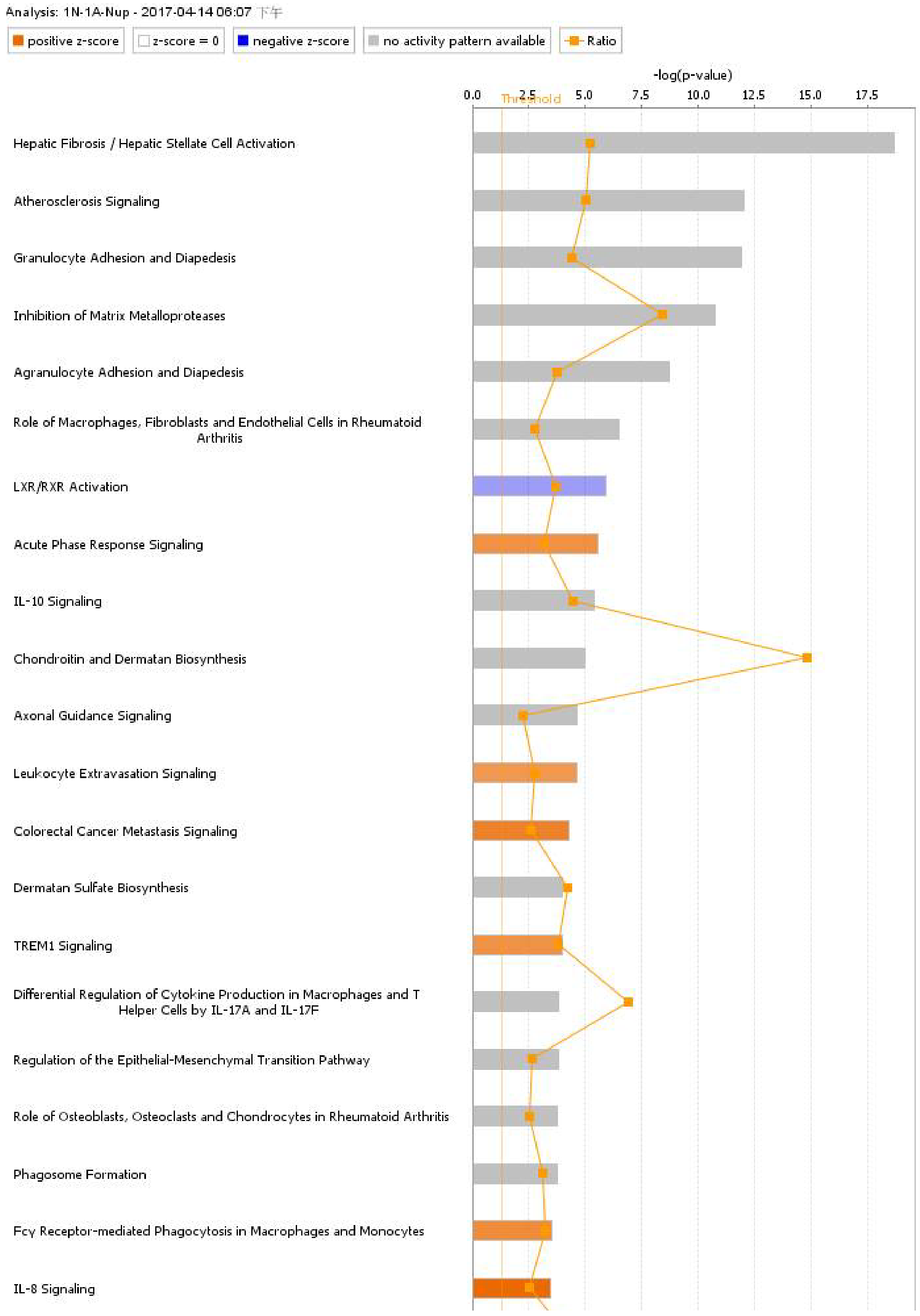

S3B Stage specific canonical pathway
week 1 N vs week 1 A condition, A upregulated

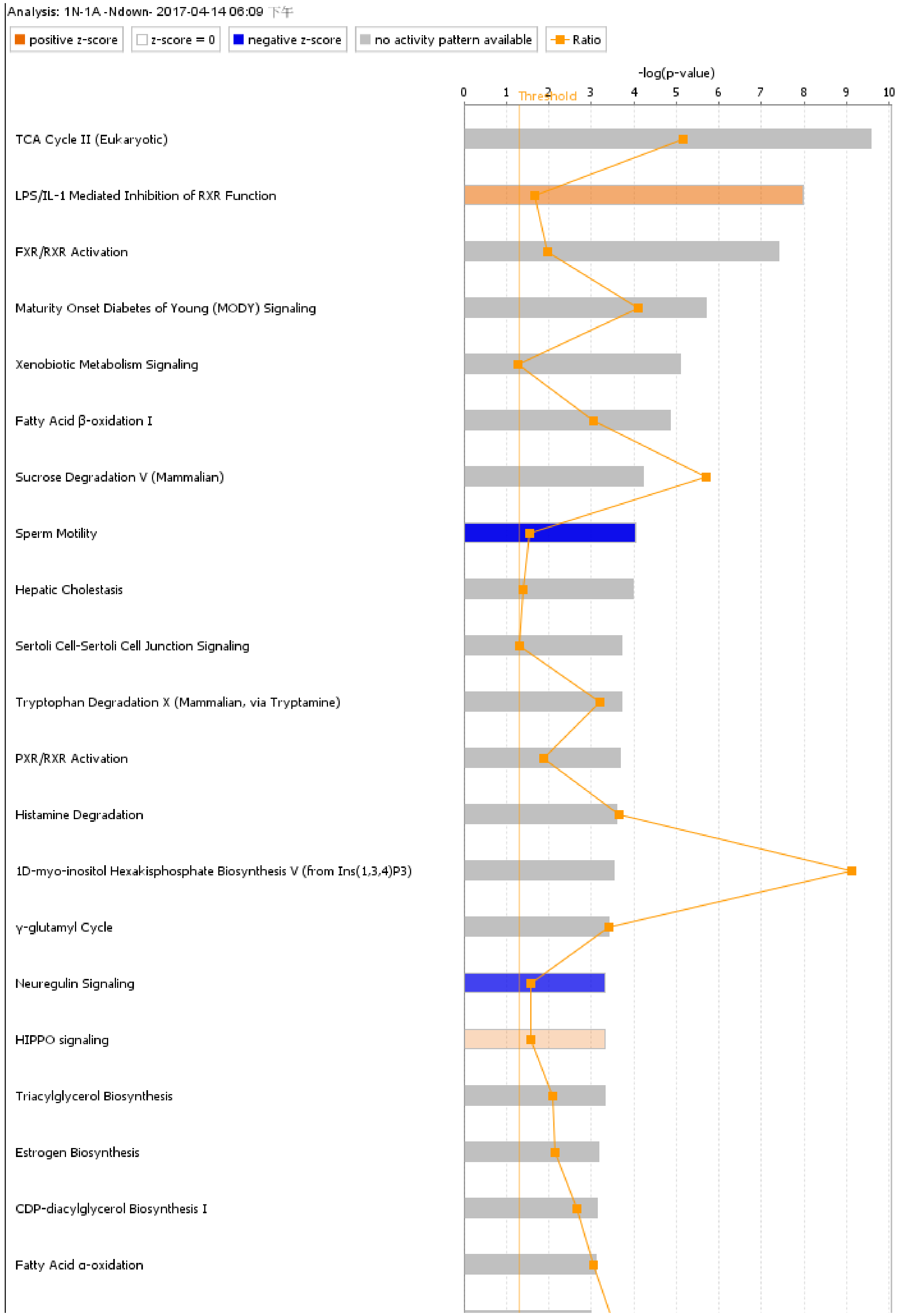

S3C Stage specific canonical pathway
week 4 N vs week 4 A condition, N upregulated

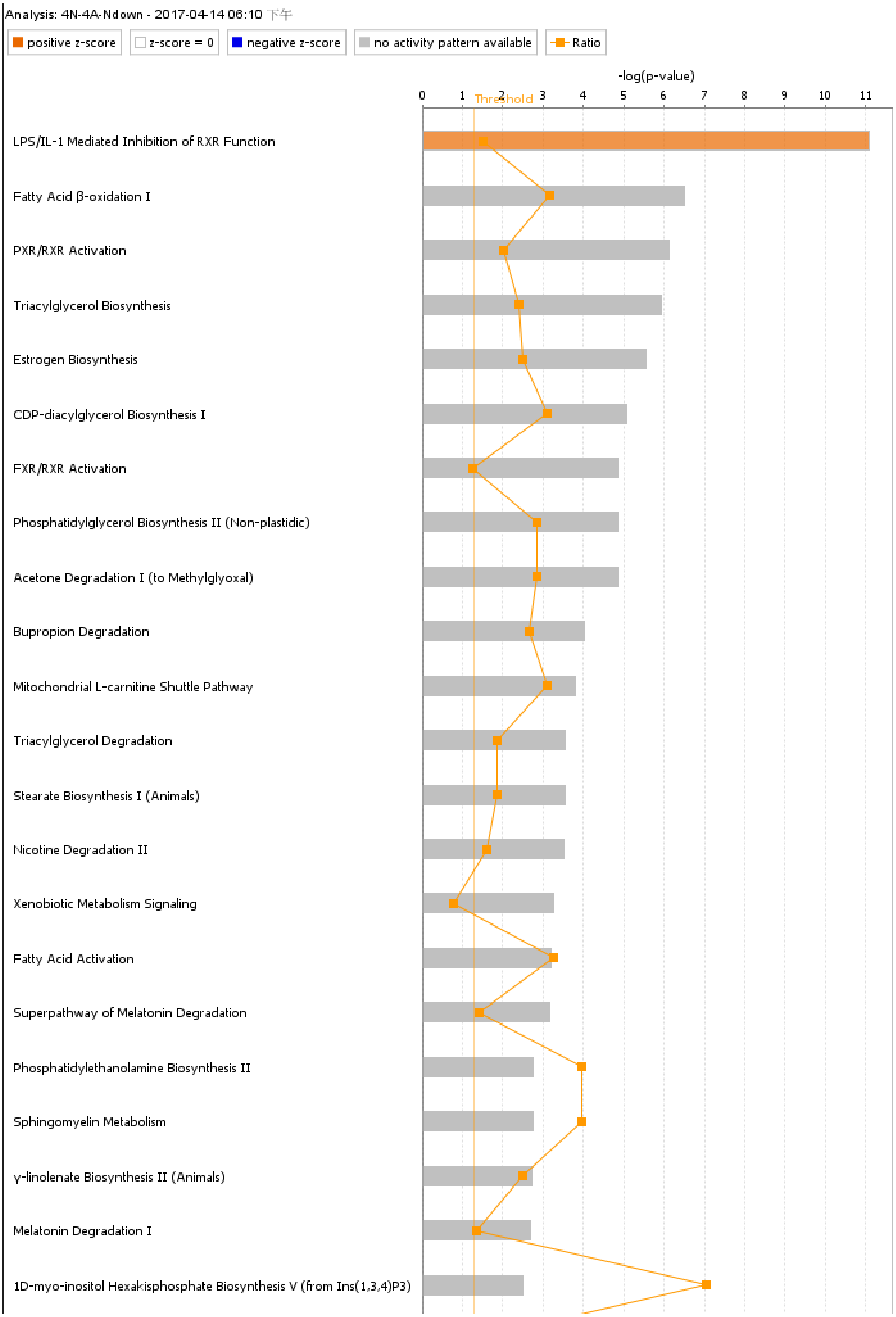

S3D Stage specific canonical pathway
week 12 N vs week 12 A condition, N upregulated

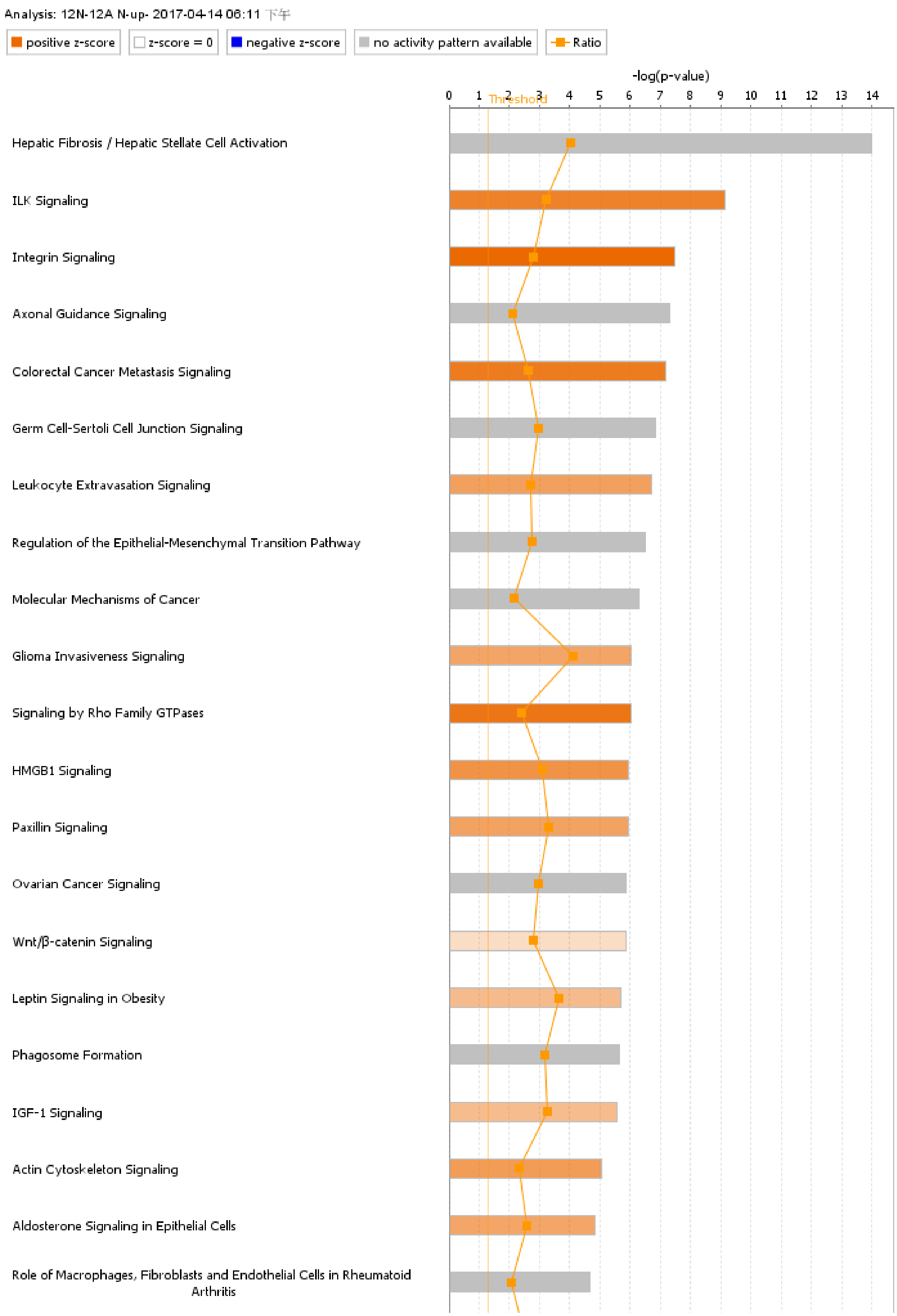

S3E Stage specific canonical pathway
week 12 N vs week 12 A condition, A upregulated

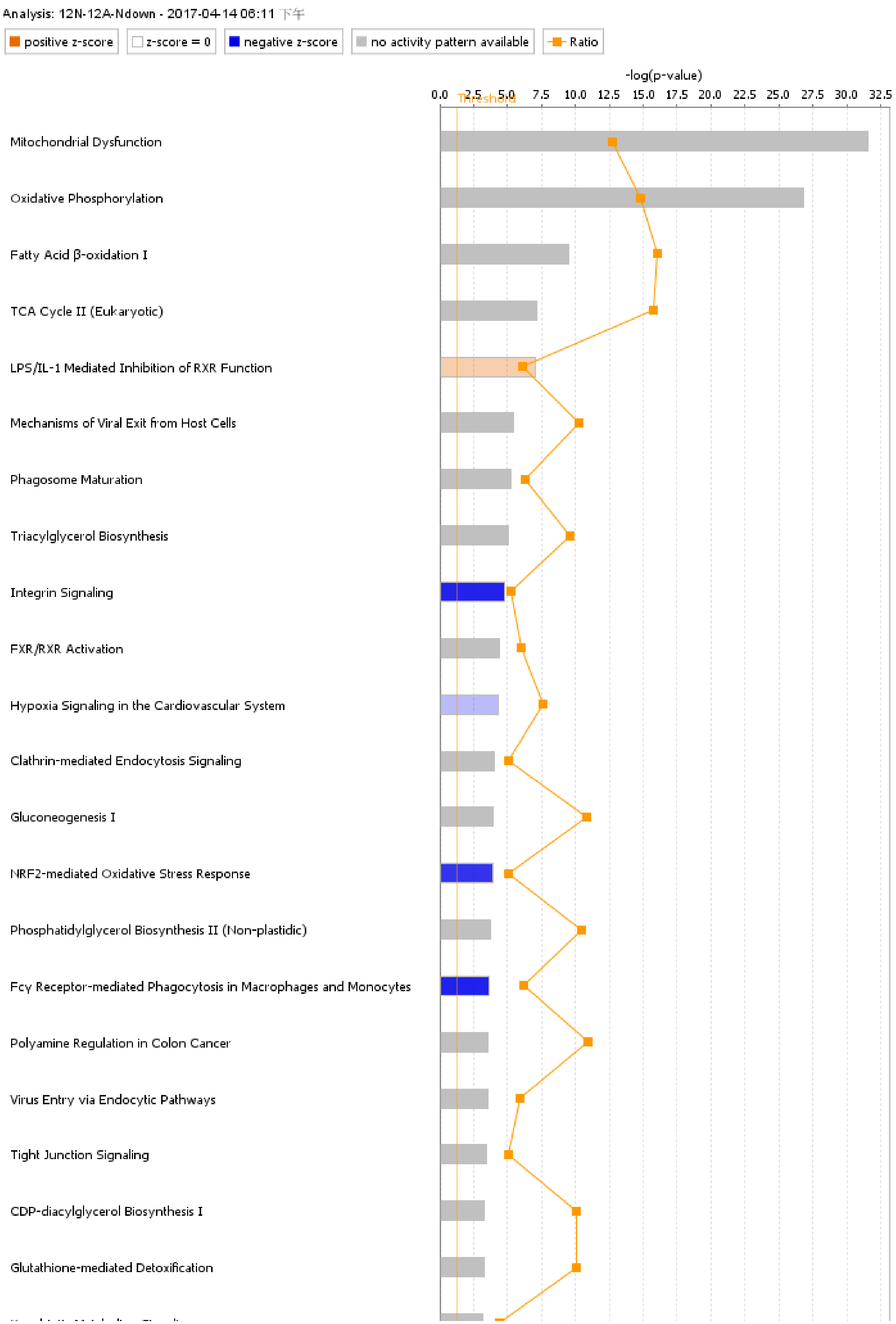

Supplement: Supplementary Materials — Suppl. Table 1: Gene Ontology analysis on week 1 “A” condition upregulated genes. Suppl. Table 2: Gene Ontology analysis on week 1 “N” condition upregulated genes. Suppl. Table 3: Oligonucleotide sequences for qPCR validation of gene expression. Suppl. Figure 1: Flowchart of this study. Suppl. Figure 2: Stage specific genomic analysis for week 1 “N” or week 1 “A” conditions. Suppl. Figure 3: Stage specific genomic analysis for 1-, 4-, and 12-week “N” vs. “A” conditions. Suppl. Dataset 1: A_stage_specific.xlsx. Suppl. Dataset 2: N_stage_specific.xlsx. Suppl. Dataset 3: NvsA.xlsx. Suppl. Dataset 4: GF_time_matrix.xlsx. [file 4037865.f1.zip › Supplementary_Info.pdf]
